# The MOGBA randomized controlled trial: Evaluation of a complex movement skill intervention for 8–12 year old children in primary school Physical Education

**DOI:** 10.1371/journal.pone.0327136

**Published:** 2025-07-28

**Authors:** Tom van Rossum, Andrew Miller, James Rudd, Johann Issartel, Jacquline D. Goodway, Donna O’Connor, Jonathan Foulkes, Jennifer Kavanagh, David Morley

**Affiliations:** 1 Centre for Child and Adolescent Physical Literacy, Carnegie School of Sport, Leeds Beckett University, Leeds, West Yorkshire, England; 2 School of Education, Faculty of Education and Arts, The University of Newcastle, Callaghan, New South Wales, Australia; 3 Norwegian School of Sport Sciences, Oslo, Norway; 4 MoveAhead, Dublin, Ireland; 5 Department of Kinesiology, College of Education, Michigan State University, East Lansing, Michigan, United States of America; 6 Faculty of Arts and Social Sciences, The University of Sydney, Sydney, New South Wales, Australia; 7 School of Sport and Exercise Sciences, Faculty of Science and Engineering, Liverpool John Moores University, Liverpool, Merseyside, England; 8 Department of Health and Human Performance, Faculty of Science and Health, Dublin City University, Dublin, Ireland; University of Tartu, ESTONIA

## Abstract

A global decline in levels of movement competence and physical activity in children presents the urgent need to look at how to reverse this trend. The Movement Oriented Games Based Assessment (MOGBA) is an intervention designed to improve children’s complex movement skills, based on principles of motor development and assessment theories. There is a positive relationship between children’s movement competence and physical activity (PA), with a further relationship established between PA and childhood obesity. This study aimed to assess how using MOGBA in PE lessons might impact primary children’s movement competence, PA, muscular fitness and self-perceptions of game and physical competence. A cluster randomized controlled trial (RCT) was conducted involving 229 children (51% girls) from nine different schools located in the north of England. The average age of participants was 9.1 years (SD = 0.21). Participants were randomized at the school level into either the MOGBA intervention group (n = 128 students) or a wait-list control group (n = 101). The MOGBA intervention was delivered over nine weeks during PE lessons by trained deliverers. The main components of the intervention included the implementation of 14 games-based activities, which were designed to support assessment within PE lessons and enhance children’s movement competence. The game-based cards also provided guidance on how to tailor activities to meet the children’s individual needs by manipulating space, effort and relationships. Pre-Post test design was employed, with participants being assessed at baseline and within 7 days post intervention. The assessment included measures of movement competence (Dragon Challenge), in-class PA (accelerometer), muscular fitness (standing long jump and plank), and perceived game and self competence (Game Play Perception Profile and Perceived Competence and Social Acceptance for Young Children). Findings show that MOGBA had a positive effect on the primary outcome of movement competence (ES: 0.18; 95%CI: −0.02, 0.38; *p* = 0.071) and a significant positive outcome (ES: 0.30; 95%CI: 0.04, 0.56; *p* = 0.025) on the way that students perceived their ability in game play. An impact was not observed on in-class PA and muscular fitness. These findings are significant as we know that increased movement competence and game self-perceptions mean children are more likely to engage with future movement, sport and physical activity opportunities. This could positively influence lifelong PA levels and promote better health. Further work should involve teachers and coaches using MOGBA to support children’s movement competence in the hopes of supporting their involvement in sport and PA. The trial is registered at the Australia New Zealand Clinical Trial Registry (ACTRN12619001320145p, 27 Sep 2019).

## Introduction

The ability to perform various movement skills (e.g., running, kicking, jumping, throwing) in a skilful manner is often defined as movement competence [1,2]. Goodway, et al. [1] state that these skills can be separated into three discrete constructs: locomotor (run, hop, jump, slide, gallop, leap); object control (strike, dribble, kick, throw, underarm roll, catch); and stability skills (non-locomotor skills such as body rolling, bending, and twisting). Collectively, these are known as fundamental movement skills (FMS). FMS are considered to be the foundational skills that enable the specialised sequences of movement required for participation in many organised and non-organised physical activities for children and adolescents [1,3].

Active participation and learning of FMS lead to the development of movement competence in children, which is positively related to increased physical activity (PA) and health-related fitness [4,5]. Moreover, developing movement competence in childhood underpins and enables successful participation in a variety of physical activities and sports later in life [6] and is a central part of promoting life-long physical literacy (PL) [7, 8]. The synergistic relationship between movement competence and PA during childhood has been shown to be mediated by self-perceptions and perceived competence [9,10] and fitness outcomes [11–13]. PA has also shown to be positively associated to cognitive skills [14] and academic attainment [15]. Considering the positive impact of movement competence and PA on children’s development, further attention to the development and evaluation of programmes that promote these outcomes is warranted. Since Whitehead’s reconceptualization of PL (7), there has been a remarkable worldwide proliferation in the use of the term and adoption of its philosophical intentions (8). As such, PL is at the forefront of national agendas to generate considerable health benefits, especially for children [16]. In England, a national consensus statement for PL (17) has recently been released to facilitate a shared understanding of PL for those working with children in all sectors.

The rate that children acquire and become competent in performing FMS is influenced by physical and biological attributes (e.g., height, genetics, maturity; strength, balance) and environmental conditions created by teachers and coaches, such as opportunities for practise, instruction, encouragement and feedback [1]. A key developmental stage within a child’s movement development is the transition from FMS to what we are defining as complex movement skills (CMS) [1,18]. CMS are mature movements that have been refined and combined in increasingly complex environments that can be used in a range of sports and PA movement settings, as children socially orientate to these environments (for example, a child catches the ball in the air and pivots to shield the ball from an opponent). Within the CMS development phase, improvements are seen in the way in which the child performs the movement skill or pattern with greater accuracy, co-ordination and control [1].

Evidence suggests that children are not achieving average levels of movement competence, globally [19,20] and in England [21], where this intervention took place. Evidence also suggests that children are dropping out of organized sport at an unprecedented rate [22,23]. The reasons for this dropout are varied and complex and include early specialization, in which a child pursues one sport and/or quits other sports to pursue one sport, which favors the development of technical skills in that sport [24]. Other factors involve a loss of focus on fun and an overemphasis on technical and tactical aspects of the game [25]. Our ability to provide positive and supportive experiences that help children progress from fundamental to complex movement skills is crucial for fostering lifelong participation in sport and physical activity.

It has been suggested that involving teachers and coaches responsible for delivering children’s sport and PA classes in the assessment of children’s movement skills would enable these practitioners to better support children’s development of FMS [21]. There is a raft of FMS assessment tools currently available, yet most of these are principally intended to be used in clinical and/or research settings [26], rather than in schools where additional factors (e.g., access to equipment; space and time constraints) can impact the feasibility of teachers using these assessments [27,28]. In recent years, a selection of FMS assessment tools has been developed with teachers and practitioners in mind as the assessor (e.g., Test of Gross Motor Development [TGMD) [29]; Canadian Assessment Movement Skill and Agility [CAMSA] [30]; Dragon Challenge [31]). The TGMD (1–3) was originally designed as a FMS assessment for both teachers and researchers consisting of locomotor and object control subscales. However, a limitation of this widely used instrument is that FMS are evaluated in isolation in simple environments that do not mirror the more complex environment of sports and games. The CAMSA and Dragon Challenge were designed to evaluate movement skills in more complex environments. The CAMSA has been designed and validated to assess children aged 8–14 years old and requires children to complete a movement-based course including seven skills that reflect *real world* abilities [32,33]. Dragon Challenge is similarly dynamic in nature in that participants are assessed over a timed obstacle course, rather than being assessed one skill at a time in isolation as seen in all other FMS assessments.

Whilst these measures move towards the assessment of a child’s movement competence in a more dynamically framed and, therefore, ecologically appropriate environment, there is still a lack of interaction with other children as they would typically experience within games [34]. To address this shortfall, a Movement Oriented Games Based Assessment (MOGBA) was designed involving an appropriate range of games-based activities and associated assessment framework to develop and assess children’s CMS competence within a dynamic and fluid environment. The study reports the evaluation of a cluster RCT using MOGBA as a 9-week intervention within physical education across primary schools in England. This trial aimed to examine whether MOGBA (a) improves children’s movement competence; (b) reduces sedentary time in school; (c), improves muscular fitness, and (d) improves self-perceptions of game and physical competence. We hypothesized that children in the MOGBA intervention, compared to those in the control group, would display more favorable changes in the variables identified above.

## Methodology

### Trial design

A cluster randomized control trial was used to assess the effectiveness of MOGBA in a primary school setting, which is discussed in detail elsewhere [35]. The trial is registered at the Australia New Zealand Clinical Trial Registry (ACTRN12619001320145p, 27 Sep 2019).

The 9-week intervention, spanning a school term, targeted children aged 9–10 years. Baseline measures, including all primary and secondary outcomes, were taken prior to the commencement of the intervention in September 2022, with post-intervention measures taken in December 2022. The use of a single site was a deviation from the original study protocol (35) in which there were originally 3 sites, (England, Ireland and Australia). The intervention began in both Ireland and Australia but were unable to continue due to school closures caused by COVID-19. After baseline measures were taken, schools were randomly allocated into either an intervention or wait-list control group. Schools allocated to the experimental group were assigned a trained deliverer to deliver the MOGBA activities across a 9-week period during PE lessons. By contrast, the wait-list control group undertook the standard PE lesson content planned by their teachers across the intervention period. Schools from the wait-list control group participated in the same intervention from October – December 2023.

### Sample size calculation and sample

A sample size calculation was completed to estimate the number of schools needed for the trial. Without accounting for clustering among schools (schools being alike and reducing the power of data), approximately 200 students were required to detect an effect of d = 0.4 at 80% power with alpha 0.05. To adjust for clustering, the following correction factor was applied (1+ (m – 1) x ICC) [23], where m = students per school and ICC = the intra-class correlation coefficient (between school variance/ between school variance + within school variance). Assumptions were based on clustering at the school level (one class recruited per school, with ~15 students per class), and a conservative ICC of 0.20 based on data from a school-based intervention [24], resulting in a correction factor of 4.0. The cluster adjusted sample was 800 students. This was to be split between 3 sites (*n* = 266 each site).

To align with the typical movement development phase associated with CMS, recruitment of participants was targeted at children 9–10 years of age. Existing university networks were used to recruit schools, and ten schools initially responded to invitation and were selected onto the trial. Representatives of each school were invited to an information event to support their decision-making in becoming involved.

**Fig 1:**
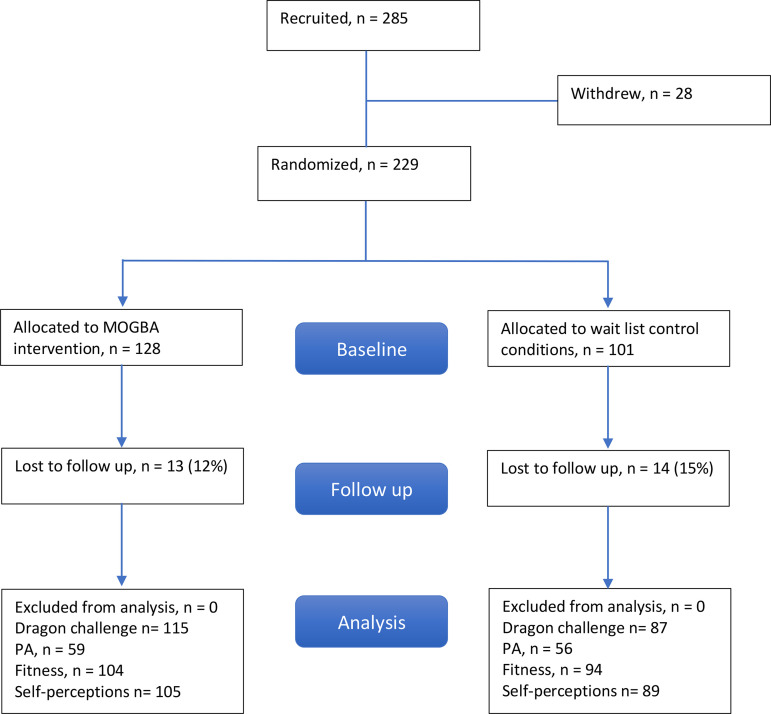
Participant recruitment and participation.

One school who initially gave consent withdrew from the study immediately prior to the baseline testing due to staffing changes, therefore, the final sample was composed of nine schools. 229 children (mean age = 9.1 years, *SD* = 0.21) were assessed at baseline, with five schools randomized to the intervention (*n* = 128) and four schools to the control (*n* = 101) group. Fig 1 shows the flow of participants through the trial. In terms of retention, measurements were obtained on 88% of the sample at post intervention. Reasons for missing data included children being absent on data collection days, losing accelerometers or not meeting the PA inclusion criteria. The available sample is clearly underpowered when compared to the planned sample. The reduction in number of sites, due to COVID-19, is one of the main reasons for the lack of statistical significance of this effect. We accept this limitation in analytical power, however, are keen to share the findings of the investigation and not contribute to publication bias in this field.

### Ethics, consent and permissions

Ethical approval for the study was obtained from the Research Ethics Committee of Leeds Beckett University (Ref: ER18592084). Recruitment of participants began Wednesday 15^th^ June 2022, with consent being received for all participants by Friday 6^th^ September 2022. Prior to the intervention, parents/carers of the participating students and the Headteacher of the school, acting as a gatekeeper, were provided with study information and provided written informed consent. Subsequently, information packs were provided to prospective participating students and their parents/carers containing information sheets and consent forms. Completed parental consent forms were collected from the class teachers and child assent forms were completed with the research team.

### Participant characteristics

The characteristics of the participant sample are presented in Table 1.

**Table 1:** Baseline characteristics of children by group.

Characteristics	Control	MOGBA	Overall
**Sample – total at baseline**			
Schools, *n*	4	5	9
Students, *n*	102	130	232
Age – years, mean (SD)	9.1 (.22)	9.1 (.21)	9.1 (.21)
Female, *n* (%)	51 (50)	66 (51)	117 (50)
British origin, *n* (%)	86 (84)	123 (95)	209 (90)
British background, *n* (%)	61 (60)	77 (59)	138 (60)
Asian background, *n* (%)	24 (24)	14 (11)	38 (16)
African background, *n* (%)	8 (8)	13 (10)	21 (9)
English as first language, *n* (%)	92 (90)	114 (88)	206 (89)
**Sample – completers**			
Students, *n* (% of baseline)	87 (86)	115 (89)	202 (88)
Age – years, mean (SD)	9.1 (.21)	9.1 (.22)	9.1 (.22)
Female, *n* (%)	42 (48)	58 (50)	100 (49)
British origin, *n* (%)	75 (86)	109 (94)	184 (91)
British background, *n* (%)	55 (63)	68 (59)	123 (61)
Asian background, *n* (%)	22 (25)	13 (11)	35 (17)
African background, *n* (%)	5 (6)	12 (10)	17 (8)
English as first language, *n* (%)	81 (93)	102 (89)	183 (91)

### Randomization

Following baseline assessments, schools at each site were ranked by their local socio-economic identifier within schooling level strata (primary/elementary) using the English indices of deprivation [36]. An individual not involved in the research project randomized schools within ranked pairs to either the MOGBA intervention or a wait list control group using a random number generator. The ninth school was initially paired with another school as an intervention school. The paired school, meant to be in the control group, withdrew at short notice preventing recruitment of a replacement school.

### MOGBA Intervention

#### MOGBA intervention deliverers training.

Relying on strategies employed from previous, similar, intervention studies [37], third-year pre-service Physical Education teachers were recruited from a university to deliver MOGBA in schools. Two, three-hour, workshops involving a mixture of MOGBA theory and practical content, were delivered by the first and last authors in a mixture of classroom and practical settings. The format of these sessions was: (i) the nature of children’s movement development, (ii) rationale, structure and purpose of MOGBA, (iii) intervention programme of activities, (iv) changing the challenge, (v) differentiating opportunities (vi) assessing and recording movement competence and (vii) using assessment to guide teaching and improve movement competence.

#### MOGBA intervention.

**Fig 2:**
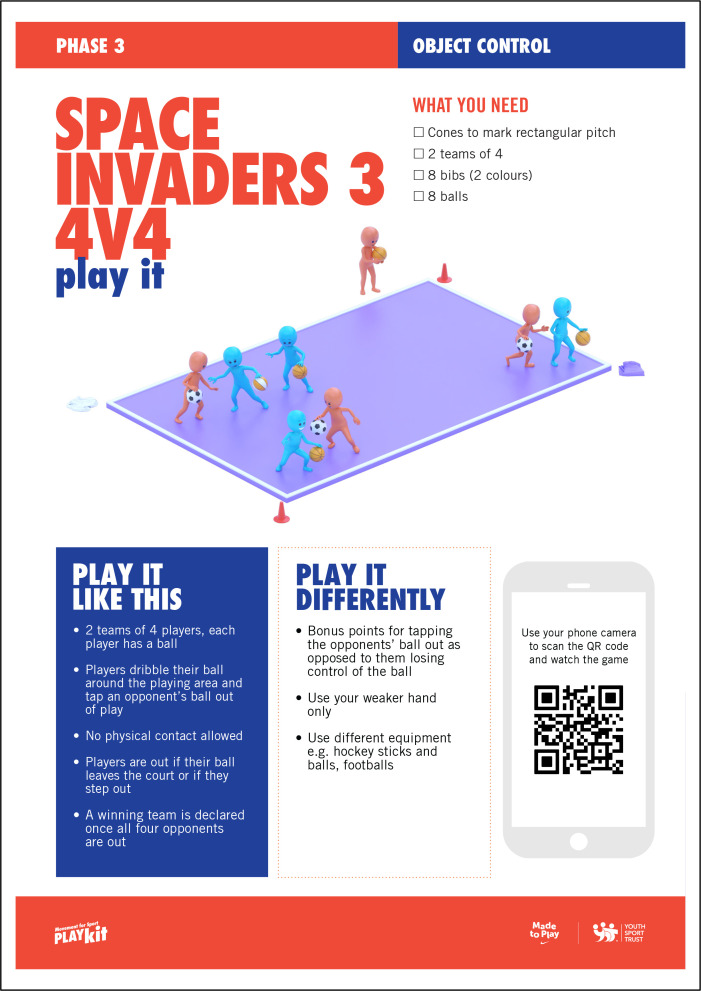
Example of a MOGBA activity (Space Invaders 4, Phase 3, Object Control) front of card ‘Play it’.

**Fig 3:**
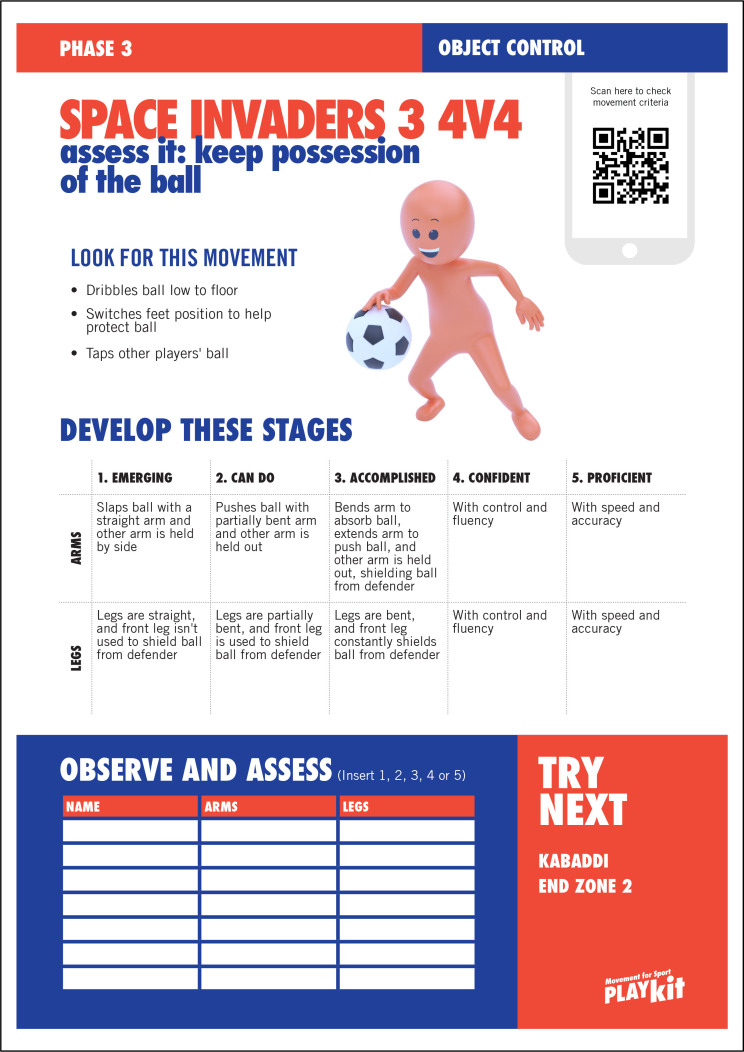
Example of a MOGBA activity (Space Invaders 4, Phase 3, Object Control) reverse of card ‘Assess it’.

The MOGBA resource is described in detail in the study protocol [35]. Briefly, MOGBA activities are designed as innovative, dynamic and fun activities that are non-sport specific and presented as three distinct phases, increasing in task complexity, perceptual-cognitive skill demand, decision making and interaction with others. MOGBA is presented as a series of 14 resource cards with the front of the card illustrating the game and additional elements to support delivery (Fig 2), whilst the reverse of the card provides an assessment framework that illustrates the movement being assessed and provides criteria for the practitioner to use to score the child’s performance using two constructs identified on the card based on observations of the head, arms, legs or body (Fig 3).

The resource also contains an ‘introductory section’ that explains the nature of children’s movement development and the purpose of MOGBA, as well as a ‘change the challenge’ section. ‘Change the challenge’ provides guidance for practitioners on how to differentiate the activity to meet the diverse needs of children in relation to Goodway, Ozmun & Gallahue’s [1] notion of Space, Effort and Relationships (SER).

MOGBA was delivered by trained deliverers in schools in one PE lesson per week over a 9-week period. Delivers used all 14 MOGBA activities based on their judgements of their class’s ability and responsiveness to the activities, ranging in categories (Stability, Object Control and Locomotion) and complexity of movement (across three phases), delivering on average 70 minutes per week (range = 60–75 minutes). The intervention deliverers were asked to modify the activity using one or more of the SER principles to challenge the children in different ways within the general construct of the game.

#### Fidelity of intervention.

The fidelity of the MOGBA intervention was evaluated by examining three big constructs of fidelity [38]: 1) *usability* (the extent to which the user can be trained to deliver MOGBA and is able to implement it), 2) *feasibility* (the extent to which MOGBA can be delivered in authentic settings of schools), and 3) *fidelity of implementation* (the extent to which MOGBA is delivered by end users in authentic contexts of school). In conducting these procedures, we addressed: 1) dose, 2) adherence, 3) exposure, 4) quality of delivery and 5) participant responsiveness, as well as addressing fidelity for the training of deliverers and the delivery of the intervention.

Fidelity was monitored through the deliverers’ adherence to a number of key determinants of successful MOGBA delivery, as agreed by an advisory panel and observed during the deliverer-led sessions in school. These determinants are listed below in Table 2. All intervention deliverers were observed by a member of the research team who was familiar with the structure and implementation procedures of MOGBA at Weeks 3 and 7 of the 9-week intervention, with sessions referenced against content indicators (see Table 2). Sessions were also judged by adherence to (1) set-up criteria (2) play the game (3) change the challenge, and (4) assessment of movement, to obtain the percentage of agreement for each of these sets of statements (E.g. lesson agreement with one of four activity-based statements = 25% activity agreement). These agreement values were used to indicate: i) if activity delivery at each time point was in line with a movement-based approach, and ii) if the fidelity of the instruction undertaken by the intervention group deliverers was in line with the intended nature of the intervention.

**Table 2:** Fidelity of MOGBA deliverer training checklist.

Core Principles	OMG a TEST	Core Practices
Clear game and Skill Introduction & Demonstration	**O**rganise a group **M**ove students into the GO position for the activity **G**ive a demonstration and a few rules simultaneously (Get it Moving!)	Deliverer creates the game in an appropriate space, following equipment and health and safety guidelines Begin lessons with a clear statement of the lesson goals (SOL) Reviews prior skills and knowledge (movement focus from previous phases) before beginning instruction Provides direct and clear description of ‘How to Play’ Checks for understanding
Targeted Elicitation	**T**ry the game- resist over instruction, increase activity exposure **E**valuate the game and student performance Uses ‘**S**TOP’ to interject with movement focus **T**ransitions students effectively between activities	Children perform the target SOL movements Deliverer checks for accurate performance and provides feedback Deliverer uses ‘change the game’ to ensure optimum engagement
Repeated Guided Practice		Deliverer provides repeated learning and practice opportunities Deliverer uses SER to differentiate activity Guides with cues (verbal, visual, physical) Provides individualised guided and varied practice opportunities Breaks down complex skills into smaller instructional units, where necessary
Deliverer Responsiveness		Provides individualized support and feedback Shows enthusiasm and is actively engaged with students Maintains class control Promotes high levels of reaching intended outcomes
Movement assessment		Deliverer identifies which children are to be assessed Accurately uses assessment criteria Affords repeated opportunities for assessment
Child Engagement		All children are actively participating, little wandering or off task behaviours Children are watching and listening when Deliverer is instructing Children are focusing their attention on the task and attempting the task as described by the teacher Children show enthusiasm for the activity tasks

Several of the authors based at the site evaluated the fidelity of intervention and also acted as mentors by observing and providing feedback to the deliverers delivering MOGBA after the observation in week 3 of the program. The observation and feedback were framed by the fidelity observational measure used in the training workshops (Table 2). This was done to support the deliverers’ understanding of the format and purpose of the designed content. This measure was also used as (1) a self-reporting mechanism for deliverers to reflect upon their delivery after every session; and (2) to form dialogue between the mentor and deliverer within the mentoring process.

### Measures and procedures

Information about children’s demographics (i.e., year and month of birth, gender, country born, cultural background and language spoken at home) were provided by the child within a questionnaire, with parents or carers providing consent. Measures were taken in a sports hall at the university of the lead author one week prior to the intervention starting (during September 2022) and within one week post intervention. PA was measured in schools during the week prior to the intervention starting and within one week post-intervention. The same instruments were used at each time point.

#### Anthropometric measurements.

Children’s height and body mass were measured with an accuracy of 0.1 cm and 0.1 kg, respectively. Height was assessed with a portable stadiometer (Leicester Height Measure, SECA, Birmingham, UK) and body mass using a digital scale (Tanita WB100-MA, Tanita Europe, The Netherlands). Measurements were taken without shoes and whilst wearing light clothing. Height and weight values were subsequently used to examine weight status through the International Obesity Task Force’s age and sex adjusted body mass index (BMI) growth-reference [39].

#### Measure of motor competence: Dragon challenge.

The primary outcome of the RCT was to improve children’s CMS as a result of exposure to the MOGBA intervention. The Dragon Challenge [40] movement assessment protocol was used to assess the movement competency of children. Dragon Challenge was selected as a dynamic and perceptual assessment of movement competence and has excellent validity and reliability [31].

Testing followed the protocol in the Dragon Challenge Manual [40]. Each child was required to complete nine activities (balance bench, core agility, wobble spot, overarm throw, basketball dribble, catch jumping, t-agility and sprint) in a continuous circuit assessing their stability, locomotor and object-control skills. The children completed the Dragon Challenge in small groups (~6) led by two field research assistants, taking approximately 20 minutes. The first research assistant was responsible for recording each trial, using a tripod-mounted video camera (Panasonic HC-V750eb-k, Malaysia), while the second was responsible for guiding each child through the Dragon Challenge, offering verbal prompts and encouragement throughout the assessment. The second research assistant was also responsible for familiarising the participants with the assessment. Prior to beginning the assessment, children were provided with a verbal explanation and single demonstration of the nine activities comprising the Dragon Challenge, in the same order that they were to be completed during the assessment. Scoring of the assessment took place at a later date via video analysis. A final Dragon Challenge score (0–54) was calculated for each participant by totalling the scores from the three assessment criteria outlined in the Dragon Challenge Manual [40], namely: technique (0–18), outcome (0–18) and time (0–18).

#### Physical activity.

All participants were asked to wear a PA monitor (accelerometer; ActiGraph GT9X Link, ActiGraph, Pensacloa, FL) on their wrist during school hours, with 100hz measurement taken every second over a period of five consecutive days to measure their activity levels during weekdays. During the monitoring period, children were asked to wear their monitors throughout the school day (09:00–15:00). Accelerometer data was reduced and analysed using ActiLife v6.0 (ActiGraph, Pensacloa, FL, USA). Valid wear time was defined as a minimum of 2 valid days, with at least 3 hours of data recorded between 09:00 h and 15:00 h (school hours). The classification of valid wear time was done following the GGIR package [41] from R software Version 4.0.2 (www.r-project.org) default option over blocks of 15 min where each block was classified as non-wear time when the standard deviation of the 60 min interval around the block was less than 13 mg in at least 2 of the 3 axes or if the value range for at least 2 of the 3 axes was less than 50 mg [41]. PA data was categorised into average minutes of daily moderate to vigorous physical activity (MVPA) for subsequent analysis.

#### Perceived self and game play competence.

To assess self-perceptions, participants were taken in small groups (~6) to a quiet space within the setting and under the supervision and guidance of a member of the research team, were asked to complete hard copies of the perceived physical competence subscale from Pictorial Scale of Perceived Competence and Social Acceptance for Young Children [42]). The PSPCSA contains four separate subscales (perceived cognitive competence, perceived physical competence, perceived peer acceptance, and perceived maternal acceptance) making up the constructs of perceived competence and social acceptance. The perceived physical competence subscale consisted of six items which were presented to the children in pictorial plates. Each of these contained two separate pictures, side by side, one of which depicted a child who was competent and the other of which depicted a child who was not so competent. The child’s task was to first select the picture which was most like themself. Then, after making that choice, the child indicated whether he or she was just a little bit like that child or a lot like that child. The range of scores for each item on the subscale was 1 (low competence and acceptance) to 4 (high competence and acceptance). This scale was developed as a valid and reliable measure for use with children [42].

Additionally, each participant completed the Game Play Perception Profile (GPPP) [43] to assess participants ability to be purposefully involved in team games and game-play skills that display perceptual-cognitive ability (defense, support & decision making). The questionnaire consists of nine statements and asks participants to respond with how much they agree with each statement on a four-point scale (1 = strongly disagree; 4 = strongly agree). The GPPP measured two distinct factors: i) game-play perception – five items reflecting ability across game themes, and ii) game self-perception – four items reflecting abilities in relation to others. A confirmatory factor analysis (CFA) was performed in R using lavaan to assess construct validity of the two-factor model in the present sample using baseline data (n = 218). Global fit indices (CFI = 0.888; RSMEA = 0.102; SRMR = 0.061) displayed moderate model fit qualities in the present sample. Inspection of modification indices indicated the question “I am good at stopping an opposition player from getting the ball” was cross loading on both factors. Removal of this question from the game-play perception factor improved model fit (CFI = 0.931; RSMEA = 0.086; SRMR = 0.051).

#### Muscular fitness.

Children’ muscular fitness was assessed using a standing long jump and plank. Participants were taken individually to a quiet space within the sports hall and under the supervision and guidance of a member of the research team to complete these measures. The standing long jump assessment followed the guidance provided in the Assessing Levels of Physical Activity Health‐Related Fitness Test Battery for Children and Adolescents (ALPHA [44]) manual. Following a single verbal and physical demonstration by a research assistant, participants were required to stand with their feet shoulder width apart, and toes just behind a line marked on the ground. Participants were then instructed to push off, jumping as far as possible whilst trying to land with their feet together and staying upright. The distance jumped was recorded by a research assistant using a tape measure, participants performed two jumps, with both attempts recorded. The plank protocol required participants to maintain a static prone position with only forearms and toes touching the floor mat they were positioned on. Participants were asked to keep their feet together with toes curled under the feet, elbows forearm distance apart, and hands clasped together against the floor mat. Participants had to maintain eye contact with their hands, a neutral spine, and a straight line from head to ankles. Participants were given one 5-s practice trial, during which a research assistant instructed the participant to adopt the proper position, followed by a 25-s period of rest. The test then began when the participant demonstrated the correct position, and a stopwatch was started. Participants were permitted to deviate from the correct position once and could continue if they immediately resumed the correct starting position. The assessment was completed on the second deviation from the correct position or if the participant did not return to the correct position after the first warning; at which point the stopwatch was stopped and the time recorded.

#### Statistical analysis.

The primary aim of the trial was to examine whether MOGBA improved children’s movement competence. PA in school, muscular fitness and self-perceptions of game and physical competence were measured as secondary variables of interest.

Statistical analyses of the primary and secondary outcomes were conducted with linear mixed models using IBM PASW Statistics 25 (SPSS Inc. Chicago, IL) software. Given the size of the sample, impacts were estimated using a completers approach to give a picture of the effects for students involved in all components of the intervention. We used pairwise deletion to make the most use of available data for each participant. Linear mixed models (LMM) were fitted to compare continuous outcomes. Group, time, and group-by-time interaction were assessed as fixed effects within the model, with covariates of gender and year level also included as fixed effects. The school a student belonged to was included as a random intercept within the model to account for the multi-level nature of the data and the nesting of children in schools. Subject (student ID) was included as a random intercept to model repeated measures at the individual level. Differences of means and 95% confidence intervals (CIs) were determined using the LMM, with alpha = 0.05 level. Effect sizes were calculated using standardized participant values (subtracting the mean score from a raw score, then dividing the result by the standard deviation) within the LMM.

## Results

### Outcome measures

The outcomes across the intervention period are presented in Table 3. The MOGBA intervention displayed a positive impact on the primary outcome (Dragon Challenge). When compared to the average change of the control group (ES: 0.18; 95%CI: −0.02, 0.38; *p* = 0.071) the effect approached statistical significance at the *p* = 0.05 level. Level 1 (student) and Level 2 (cluster) residuals displayed compliance to normality assumptions (Shapiro-Wilk: Level 1 = 0.996; df: 404; p = 0.519; Level 2 = 0.839; df: 9; p = 0.057). With regard to participants lost-to-follow-up, Dragon Challenge average scores at baseline were marginally lower among non-completers (Control: Lost = 27.87; Complete = 31.70; MOGBA: Lost = 29.33; Complete = 31.29), with marginally greater female representation among participants lost in the control condition (Lost: 60%; Complete 48%). To test the sensitivity of the primary outcome analysis to missing data, we assessed the final model using all available data. This analysis displayed a marginally lower effect size estimate (ES: 0.16; 95%CI: −0.03, 0.35; *p* = 0.104), demonstrating minimal impact of missing data on the primary outcome. Game perceptions (referencing self) displayed positive change among the MOGBA group, with significant positive effects when compared to the control condition (ES: 0.30; 95%CI: 0.04, 0.56; *p* = 0.025). The MOGBA intervention appeared to have no impact on PA in school, with trivial difference observed between conditions across the intervention period (ES: −0.07; 95%CI: −0.41, 0.26; *p* = 0.664). Additional analysis of outcome data for participants who did not complete the PA measures at follow-up is provided in a Table as supplementary materials. The MOGBA group demonstrated declining results for the standing jump and plank tests, which led to negative effect sizes when compared to the control condition. Among self-perception measures, the intervention demonstrated no impact on physical self-perception or game self-perception (referencing other students), with no difference when comparing conditions.

**Table 3:** MOGBA intervention effects.

Outcome	n	Baseline, mean (SD)	Baseline difference as effect size (*d*)	Lost-to-follow-up (%)	Mean change from baseline (95% CI)	Adjusted mean difference (95% CI)^a^	Adjusted effect size *d* (95% CI)^a^	Group x time *p*
**Dragon Challenge**								
MOGBA	115	31.29 (5.98)	0.072	12	3.09* (2.30, 3.87)	1.10 (−0.96, 2.29)	0.18 (−0.02, 0.38)	0.071
Control	87	31.70 (5.27)	Reference	15	1.99* (1.09, 2.89)	Reference	Reference	
**Weekday PA**								
MOGBA	59	25.06 (9.39)	0.191	40	−0.41 (−2.75, 1.94)	−0.74 (−4.10, 2.62)	−0.07 (−0.41, 0.26)	0.664
Control	56	26.85 (9.39)	Reference	33	0.33 (−2.07, 2.74)	Reference	Reference	
**Standing-jump**								
MOGBA	104	90.49 (21.44)	−0.358	11	−5.34 (−11.9, 1.21)	−9.33 (−18.91, 0.25)	−0.33 (−0.66, 0.01)	0.056
Control	94	83.45 (16.78)	Reference	8	3.99 (−3.07, 11.04)	Reference	Reference	
**Plank**								
MOGBA	104	106.5 (90.91)	−0.313	11	−27.03* (−39.94, −14.12)	−20.88 (−39.78, −1.98)	−0.29 (−0.54, −0.03)	0.031
Control	94	81.62 (62.17)	Reference	8	−6.15 (−19.96, 7.66)	Reference	Reference	
**Physical self-perception**								
MOGBA	105	2.46 (0.42)	−0.304	18	0.05 (−0.05, 0.15)	−0.06 (−0.21, 0.09)	−0.15 (−0.51, 0.20)	0.405
Control	89	2.32 (0.50)	Reference	12	0.11 (0.01, 0.22)	Reference	Reference	
**Game perception**								
MOGBA	105	2.89 (0.64)	0.127	9	0.12* (0.01, 0.22)	0.18 (0.02, 0.34)	0.30 (0.04, 0.56)	0.025
Control	89	2.97 (0.62)	Reference	12	−0.07 (−0.19, 0.05)	Reference	Reference	
**Game perception (others)**								
MOGBA	105	2.81 (0.60)	0.083	9	0.00 (−0.11, 0.12)	0.05 (−0.12, 0.23)	0.09 (−0.19, 0.38)	0.532
Control	89	2.86 (0.60)	Reference	12	−0.05 (−0.18, 0.07)	Reference	Reference	

* significant at *p* < 0.05

^a^Between-group difference of change score (intervention change minus control change)

### Fidelity of intervention

Coding of lesson observations conducted in weeks 3 and 7 of the intervention (see Table 4) illustrate that, overall, there was good adherence of MOGBA measured in line with key determinants of successful delivery of the intervention. Movement assessment, references to quality of movement and the game, and individual feedback being offered were seen in less than half of the lessons observed in weeks 3 and 7, with no notable change between the weeks. Class engagement during the activities and class control by the deliverer increased from Week 3 to Week 7.

**Table 4:** Fidelity of intervention.

	Visit in week 3 (%)	Visit in week 7 (%)
Organize groups	100	100
Move to GO position	100	100
Give demonstration and rules simultaneously	87	69
Try (within 4mins)	73	94
Set-up (as described on card)	93	81
Play the Game (as described)	100	87
Change the Game (as described)	60	50
Movement assessment possible	53	44
Reference made to quality of movement	20	25
Reference made to quality of the game	47	44
Individual movement feedback offered (Phase 1 – wk 3 fidelity check)	20	19
>75% of class engaged during activity	87	94
Maintains class control (Adherence to teacher instruction, address off-task behavior, stops dangerous play)	80	87

% = total amount of adherence observed at all MOGBA delivery sites

## Discussion

This trial aimed to examine whether MOGBA (a) improves children’s movement competence (Dragon Challenge); (b) reduces sedentary time during the school day (accelerometers); (c), improves muscular fitness (plank and standing long jump), and (e) improves self-perceptions of game and physical competence (Game Play Perception Profile and Perceived Competence and Social Acceptance for Young Children).

Importantly, our study suggests that MOGBA achieves its primary aim by positively impacting on children’s movement competence. Children in the MOGBA intervention group displayed a greater increase in score for the Dragon Challenge (MOGBA group increased 3.09 from baseline, compared with an increase of 1.99 from the mean for the control group) across the 9 week intervention. However, there was no significant difference between scores in the intervention and control groups. While MOGBA may have contributed to improvements in movement competence, the evidence is not strong enough to conclusively demonstrate a greater effect compared to the control. Future studies with a larger sample size and additional subgroup analysis would help to clarify these findings. The potential for MOGBA to positively impact on movement competence aligns with the results of the PLUNGE intervention [45], which also showed improvements in FMS after a short intervention period within PE lessons. This finding is especially notable given the limited external input and relatively short duration of the intervention, both of which have positive implications for scalability.

Recognizing the positive synergistic relationship between movement competence and PA levels [22], further research is needed to better understand the extent to which MOGBA may influence movement competence and its relationship to PA during childhood and adolescence. Mid- to late- childhood is recognized as a key stage for children’s movement development transitioning from FMS to CMS [1]. There is a major gap in the literature with little literature investigating the complex movement skills that occur after FMS have been developed. The increasing complexity and challenge of how FMS are combined and performed in the MOGBA games fosters an environment for these movements to be refined and developed into CMS, which are necessary to take part in a range of sports and PA movement settings [1].

This study also found that MOGBA had a significant positive outcome on the way that students perceived their ability in game play (0.12* mean change from baseline). We would point to the progressive, de-constructed nature of the MOGBA games in comparison to sports-based approaches as one of the reasons why children’s self-perceptions of their ability to play games improved. Taken together the MOGBA intervention represents a positive step forward for strategies to enhance movement competence and game play confidence. The MOGBA assessment framework, employed as an assessment for learning tool, offered valuable feedback to deliverers that could be used for benchmarking and monitoring of children’s movement competence [46,47]. This approach differs from traditional ‘assessment of learning,’ which often involves summative grading. Instead, it enables participants to directly observe their progress and recognize areas for improvement using movement-based criteria. This method could partly explain the observed improvements in both movement competence and game self-perceptions, as the dynamic, game-based assessment framework of MOGBA contrasts with more traditional, isolated movement assessments. Greater adherence by the deliverers to provide feedback on the quality of the movement (see Table 4) could have further improved children’s movement competence as a result of the intervention. This positive effect on both movement competence and perceived movement competence is crucial because improved self-perception in this area increases the likelihood of children engaging in future PA and sports [8]. By demonstrating the potential to positively impact children’s self-perceptions and movement competence, MOGBA appears to be an effective intervention for developing constructs of PL [7,17].

The present study demonstrated no significant change (statistically and practically) in PA among the MOGBA (−0.41 minutes/day mean change from baseline) or control (0.33 minutes/day mean change from baseline) groups. This finding is unsurprising as a recent systematic review [48] found that school-based PA interventions can result in little to no increase in MVPA. School-based PA interventions that have positively impacted MVPA were implemented over 1 year [49] and 2 years [50] with both interventions being embedded across the school day, not just in PE lessons. This aligns with the conclusions of a review conducted by van Sluijs and colleagues [51], that quantity of delivery is essential for a school-based PA intervention to be successful. Therefore, a longer intervention period or more frequent exposure to MOGBA (e.g., at lunch times or after school) could have resulted in a different response. Furthermore, children have little control over their PA for much of the school day, with the type of activity and its duration being controlled by the teacher, so getting PA changes within schools is more challenging.

It is difficult to determine what happened with the control group that led to greater improvements in standing long jump and lower decline in plank scores than the MOGBA group (leading to negative effect sizes for the intervention). Participants within the control group may have been engaged in strength-based activities within the lessons delivered during the intervention period resulting in improvements to explosive strength. Furthermore, MOGBA does not overtly emphasize this aspect of athletic development, thus de-training could have occurred over 9 weeks [52]. Considering the synergistic relationship between muscular fitness and movement competence [53], further exploration of this finding is warranted to understand the potential for MOGBA to negatively impact on explosive strength as this is an important determinant of fitness [52].

It is noteworthy that our findings show that muscular fitness outcomes were highly divergent at base line. It has been reported that completing a warm-up immediately prior to testing can improve standing long jump performance [54], thus the ordering of measurements taken in the data collection setting could have influenced these results. Despite standardized approaches being used for data collection at pre- and post- intervention, we are also unable to rule out measurement error as an explanation for these unexpected results. These differences render the drawing of inference from these data problematic.

### Strengths and limitations

The study strengths are the cluster RCT design and the positive impact of MOGBA delivered in primary school PE on children’s movement competence and game play perceptions. MOGBA has shown positive results comparable to other programs aimed at promoting movement skills and PA (PLUNGE [45]), or promoting PA specifically in PE lessons (SAMPLE PE [37]). An advantage of MOGBA over these existing programs is that it uses a train-the-trainer model, rather than the teacher delivering it themselves, which has potential to be more scalable for use in a wider number of settings. This is particularly important as school-based interventions to develop FMS have potential to have greater impact due to children having more sustained time in school [51,55].

There were, however, several limitations that warrant consideration. Firstly, the reduction in the number of sites due to COVID-19 and, therefore, a reduction in the number of participants negatively impacted the effect size. Secondly, the reduction in sites also led to inability to explore global differences in the effectiveness of MOGBA as was intended in the original study design. Thirdly, missing student data is almost exclusively due to absence at follow-up data collection resulting from student illness. This is considered a secondary, lingering effect of the disruption caused to the study by COVID-19. However, overall attrition (12%) and differential attrition (3%) for the primary outcome (movement competence) were considered low in accordance to the What Works Clearinghouse (WWC), established by the U.S. Department of Education’s Institute of Education Sciences (see http://ies.ed.gov/ncee/wwc/). The majority of secondary outcomes follow a similar pattern for available data (Table 3), with the exception of the accelerometer data, which saw a loss of ~40% of matched data at follow-up due to the logistics of data collection involving accelerometers in schools. Combined with 7% differential attrition between groups, attrition for the PA data was considered “high”. Finally, observations of the control group were not conducted, leaving uncertainty around the exact content and dose delivered to these participants.

Although using external deliverers, as opposed to teachers, to deliver the intervention was appropriate within this efficacy trial, there is a need to evaluate the effectiveness of MOGBA when scaled to primary teachers as the deliverers [56]. Ensuring that appropriate CPD is designed, delivered and evaluated is necessary to ensure that the integrity and fidelity of MOGBA is maintained throughout any scaling process. Recognizing the relatively low adherence to fidelity measures by the delivery team, establishing better ways of supporting high quality delivery should be a focus for future implementation.

## Conclusion

To the authors’ knowledge, MOGBA is the first CMS intervention intended to be used in education and sport settings for children aged 8–12 years. MOGBA is also the first intervention that combines delivery and movement competence assessment protocols as well as conducting the assessment within a game situation. Results indicate that MOGBA positively impacts on children’s movement competence and game play perceptions. Continued research should address the initial aim of the registered trial through the establishment of a global RCT, so that we can begin to assess the effectiveness of MOGBA in affecting children’s movement competence, PA, fitness and self-perceptions across countries.

MOGBA shows promise as a tool to support children on a positive PL journey by “building the skills, knowledge and behaviours needed to help us lead active lives” [57]. Albeit, further research is needed to explore the extent to which MOGBA may promote PL. Further work also needs to be undertaken involving PE teachers and other specialist practitioners who could use MOGBA to support children’s involvement in sport and PA. Sustained training and support to deliverers throughout the period of the intervention should be considered to amplify its impact [58]. Involving parents and extending the intervention to the home could further strengthen its effectiveness [56].

## Supporting information

S1 File CONSORT checklist.
(DOCX)

S2 File Physical activity outcome - Differences among analytic group.
(DOCX)

S3 File Study protocol.
(PDF)
