# Supplementary material for: The MOGBA randomized controlled trial: Evaluation of a complex movement skill intervention for 8–12 year old children in primary school Physical Education
Source: PLoS One. 2025 Jul 28;20(7):e0327136. doi: 10.1371/journal.pone.0327136 (PMC12303325; doi:10.1371/journal.pone.0327136)
Supplement: S2 File — Physical activity outcome - Differences among analytic group. (DOCX) [file pone.0327136.s002.docx]

**MOGBA – supplementary table**

Physical activity outcome - Differences among analytic group

| **Characteristics** |  | **Control** |  |  | **MOGBA** |  |
| --- | --- | --- | --- | --- | --- | --- |
|  | **Analysis group** | **%** | **N** |  | **%** | **N** |
| Female, % | Complete | 50 | 28 |  | 51 | 30 |
|  | Lost-to-follow-up | 43 | 13 |  | 54 | 25 |
|  | Missing | 63 | 10 |  | 44 | 11 |
|  |  | **Mean (SD)** | **N** |  | **Mean (SD)** | **N** |
| Age - years, mean (SD) | Complete | 9.0 (0.1) | 56 |  | 9.0 (0.2) | 59 |
|  | Lost-to-follow-up | 9.1 (0.3) | 30 |  | 9.0 (0.2) | 46 |
|  | Missing* | 9.1 (0.3) | 16 |  | 9.1 (0.3) | 25 |
| Dragon challenge, mean (SD) | Complete | 32.4 (5.2) | 56 |  | 32.8 (5.3) | 59 |
|  | Lost-to-follow-up | 30.8 (5.8) | 30 |  | 28.9 (6.6) | 46 |
|  | Missing* | 27.6 (7.7) | 16 |  | 30.9 (5.4) | 25 |
| Weekday PA - minutes, mean (SD) | Complete | 26.9 (9.4) | 56 |  | 25.0 (9.4) | 59 |
|  | Lost-to-follow-up | 21.8 (8.9) | 30 |  | 27.7 (10.5) | 46 |
|  | Missing* | N/A |  |  | N/A |  |
| Game perception, mean (SD) | Complete | 2.9 (0.7) | 56 |  | 2.9 (0.7) | 59 |
|  | Lost-to-follow-up | 2.9 (0.5) | 30 |  | 2.9 (0.7) | 44 |
|  | Missing* | 3.0 (0.6) | 15 |  | 2.9 (0.5) | 24 |

*These are participants with partial completion of measurements but no PA data

The date in the supplementary table illustrates that there was a slightly greater proportion of females that did not complete the PA measurements post-intervention. There was very little difference in Dragon challenge scores of completers of PA measurement and those lost to follow up. Participants lost-to-follow-up in the MOGBA group had marginally higher baseline PA.
