# Supplementary material for: The MOGBA randomized controlled trial: Evaluation of a complex movement skill intervention for 8–12 year old children in primary school Physical Education
Source: PLoS One. 2025 Jul 28;20(7):e0327136. doi: 10.1371/journal.pone.0327136 (PMC12303325; doi:10.1371/journal.pone.0327136)
Supplement: S3 File — Study protocol. (PDF) [file pone.0327136.s003.pdf]

PLOS ONE

REGISTERED REPORT PROTOCOL

Rationale and study protocol for the Movement Oriented Games Based Assessment (MOGBA) cluster randomized controlled trial: A complex movement skill intervention for 8–12 year old children within 'Made to Play'

David Morley[1☯*], **James Rudd**[2☯], **Johann Issartel**[3☯], **Jackie Goodway**[4☯], **Donna O'Connor**[5‡], **Jonathon Foulkes**[2‡], **Mark Babic**[6‡], **Jennifer Kavanagh**[3‡], **Andrew Miller**[6☯]

1 Discipline of Sport and Exercise Science, La Trobe University, Melbourne, Victoria, Australia, **2** School of Sport and Exercise Sciences, Faculty of Science and Engineering, Liverpool John Moores University, Liverpool, Merseyside, England, **3** Department of Health and Human Performance, Faculty of Science and Health, Dublin City University, Dublin, Ireland, **4** Department of Human Sciences, College of Education and Human Ecology, The Ohio State University, Columbus, Ohio, United States of America, **5** Sydney School of Education and Social Work, Faculty of Arts and Social Sciences, The University of Sydney, Sydney, New South Wales, Australia, **6** School of Education, Faculty of Education and Arts, The University of Newcastle, Callaghan, New South Wales, Australia

☯ These authors contributed equally to this work.
‡ These authors also contributed equally to this work.
* dave.morley@latrobe.edu.au

Check for updates

This is a Registered Report and may have an associated publication; please check the article page on the journal site for any related articles.

OPEN ACCESS

Citation: Morley D, Rudd J, Issartel J, Goodway J, O'Connor D, Foulkes J, et al. (2021) Rationale and study protocol for the Movement Oriented Games Based Assessment (MOGBA) cluster randomized controlled trial: A complex movement skill intervention for 8–12 year old children within 'Made to Play'. PLoS ONE 16(6): e0253747. https://doi.org/10.1371/journal.pone.0253747

Editor: Francisco Javier Huertas-Delgado, La Inmaculada Teacher Training Centre (University of Granada), SPAIN

Received: October 25, 2020

Accepted: May 14, 2021

Published: June 24, 2021

Copyright: © 2021 Morley et al. This is an open access article distributed under the terms of the Creative Commons Attribution License, which permits unrestricted use, distribution, and reproduction in any medium, provided the original author and source are credited.

Data Availability Statement: All relevant data from this study will be made available upon study completion.

Funding: The Youth Sport Trust (https://www.youthsporttrust.org) provided funding for this

Abstract

There is a positive relationship between children's movement competence and physical activity, with a further relationship established between physical activity and childhood obesity. The Movement Oriented Games Based Assessment (MOGBA) is a delivery and assessment intervention designed to improve children's complex movement skills, based on principles of motor development and assessment theories. MOGBA aims to improve children's movement competence, physical fitness and self-perceptions (physical and game) and increase children's moderate-to-vigorous physical activity (MVPA). MOGBA is to be used in the 'Made to Play' initiative, involving 105 sports and activity programs across 21 countries, involving over 25 million children. A multi-site cluster randomized controlled trial will take place across three global sites (UK, Ireland and Australia). Each site will recruit eight primary schools (four experiment, four control) with each school providing two separate classes of children from age ranges 8–12 years (Site n = ~300, total n = 904). After baseline assessments, schools will be randomly allocated to an experimental or wait-list control group. Following two half-day workshops, trained facilitators will deliver the MOGBA intervention for 9 weeks. The main intervention components include delivery of 14 games-based activities with associated assessments of children's movement and differentiation to meet children's needs by manipulating space, effort and relationships. The primary outcome

project, by way of supporting the costs of resource development and transcription services and had no say in the research design. The Youth Sport Trust received funding for the project from Nike. None of the authors have any financial benefit arising from the research.

Competing interests: The Youth Sport Trust provided funding for this project, by way of supporting the costs of resource development and transcription services and had no say in the research design. The lead author is working as a consultant (project lead) for the Youth Sport Trust and Nike on the project. This does not alter our adherence to PLOS ONE policies on sharing data and materials. None of the authors have any financial benefit arising from the research.

of the trial is to improve children's' movement competence (The Dragon Challenge), with secondary outcomes of improving children's' in-activity and leisure-time MVPA (5-day accelerometer), physical fitness (standing long jump and push ups) and self-perceptions (physical and game). Data will be analysed using multilevel modelling approaches. The MOGBA intervention has been designed to improve children's movement competence and scalable interventions based on MOGBA could be applied across programs within the Made to Play initiative, globally. The trial is registered at the Australia New Zealand Clinical Trial Registry (ACTRN12619001320145p, 27 Sep 2019).

Introduction

The ability to perform various movement skills (e.g. running, kicking, jumping, throwing) in a skilful manner is often defined as movement competence [1, 2]. Goodway, et al. [1] state that these skills can be separated into three discrete constructs: locomotor (run, hop, jump, slide, gallop, leap); object control (strike, dribble, kick, throw, underarm roll, catch); and stability skills (non-locomotor skills such as body rolling, bending, and twisting). Collectively, these are known as Fundamental Movement Skills (FMS) and are considered to be the foundation skills that enable the specialised sequences of movement required for participation in many organised and non-organised physical activities for children and adolescents [1, 3]. Furthermore, a positive relationship has been established between children's participation in physical activity (PA) and movement competence [4–7] and PA has been recognized as supporting broader constructs of children's development within the cognitive, psychological and emotional domains [8, 9].

A key developmental stage within a child's movement development is the transition from FMS to what we are defining as Complex Movement Skills (CMS) [1, 10]. CMS are mature movements that have been refined and combined in increasingly complex environments that can be used in a range of sports and physical activity movement settings, as children socially orientate to these environments. Within the CMS development phase, improvements are seen in the way in which the child performs the movement skill or pattern with greater accuracy, co-ordination and control [1]. The rate that children acquire and become competent in performing FMS is influenced by physical attributes (e.g. height, genetics, maturity) and environmental conditions created by teachers and coaches, such as opportunities for practise, instruction, encouragement and feedback [11].

There is a raft of FMS assessment frameworks that have been validated, refined and used cross-sectionally to understand the movement competence of children [12]. Typically, movement assessments have been designed to assess children at the FMS stage of development and are intended for use by clinicians and researchers, deeming them unsuitable for use by teachers in a practical environment [13, 14]. It has been suggested that involving practitioners responsible for delivering children's sport and PA classes in the assessment of children's movement skills would enable practitioners to better support children's development of FMS [15]. In recent years, a selection of FMS assessment tools has been developed with teachers and practitioners in mind as the assessor (e.g. Canadian Assessment Movement Skill and Agility [CAMSA] [16]; Dragon Challenge [17]). CAMSA has been designed and validated to assess children aged 8–14 years old and requires children to complete a movement-based course including seven skills that reflect *real world* abilities [18, 19]. Dragon Challenge is similarly dynamic in nature in that participants are assessed over a timed obstacle course, rather than being assessed one skill at a time in isolation as seen in all other FMS assessments.

Whilst these measures move towards the assessment of a child's movement competence in a more dynamically framed and, therefore, ecologically appropriate environment, there is still a lack of interaction with other children as they would typically experience within games [20]. To address this shortfall, a Movement Oriented Games Based Assessment (MOGBA) has been designed to provide an appropriate range of games-based activities and associated assessment framework to develop and assess children's CMS competence within a dynamic and fluid environment. The aim of this paper is to outline the rationale and study protocol for a MOGBA cluster randomized controlled trial.

The Made to Play (MTP) context

The MTP initiative aims to support 105 programs across 21 countries in providing 'opportunities to get children moving so that they can lead happier, healthier and more successful lives' [21]. To achieve this, one of their objectives is to support coaches within MTP programs to develop the movement competence of children aged 8–12 years by providing a delivery and assessment framework. The programs supported by MTP are wide-ranging, containing the full spectrum of individual (e.g. running, skateboarding) and team (e.g. basketball, soccer) sports. MTP programs are delivered in a range of settings (e.g. schools, recreational centres, sports clubs) using varying models and patterns of delivery in both participation and performance domains, with group sizes typically between 15 and 30 children.

Materials and methods

Ethics

Sheffield Hallam University Ethics Committee granted ethical approval (ER18592084), with extension to 2022. The University of Newcastle, Australia, has also granted ethics approval (H-2020-0066). The trial is registered at the Australia New Zealand Clinical Trial Registry (ACTRN12619001320145p, 27 Sep 2019). Written consent will be obtained from participants.

Trial design

A cluster randomized control trial will be used to assess the effectiveness of MOGBA in a school setting, as this is perceived to be an ideal setting for the promotion of movement competence and PA [22]. Another potential option available was to establish the trial within MTP programs, as clusters. It was felt that, due to the eclectic and disparate nature of these programs, controlling and accounting for the variability both within intervention and control groups would be more problematic than a school setting. It is intended to use the findings of this trial to inform the scalability of MOGBA across MTP programs.

Through the trial we aim to examine whether MOGBA (a) improves children's movement competence; (b) increases Moderate to Vigorous Physical Activity (MVPA during PE lessons, (c) increases MVPA time in school (d), improves physical fitness, (e) improves self-perceptions of game and physical competence. We hypothesize that children in the MOGBA intervention, compared to those in the control group, will display more favorable changes in movement competence, physical activity, physical fitness, physical self-perceptions and game perceptions.

The 9-week intervention will be delivered during the school day and target children in the school years containing the age ranges 8/9, 9/10, 10/11, 11/12 years. Baseline measures, including all primary and secondary outcomes, will be taken prior to the commencement of the intervention, which will be staggered across the sites to prevent clashes with key educational milestones within the respective countries. This is a deviation from the original study protocol in which there was originally 4 sites, including the USA, which was subsequently reduced to 3

sites as a result of Covid-19 restrictions on accessing schools. After baseline measures are taken, schools will be randomly allocated into either an intervention or wait-list control group. Schools allocated to the experimental group will be assigned a facilitator to deliver the 14 MOGBA activities across a 9-week period.

By contrast, the wait-list control group undertakes the standard content planned by their teachers across the intervention period. This content will vary from country to country and will also be subject to change based on the seasonality of delivery across the RCT. As each site has its own control group, it is not expected that heterogeneity among the countries will introduce bias. Additionally, as the countries involved have relatively similar physical education programs expressed through the development of movement skills with transference into game and sport based activities, the differences related to individual sites is likely minimal. All countries will follow some sort of Physical Education curriculum, whether that is determined at national, provincial or regional level. All primary and secondary outcomes will be assessed at 3-month post-test with the exception of in-school physical activity, which will be assessed between weeks 7–9 of the intervention period to assess the impact of the program on student in-school MVPA. Schools from the wait-list control group will receive the MOGBA program after the completion of post-test assessments.

Sample size calculation

A sample size calculation was completed to estimate the number of schools needed for the trial. Sampling for a two-group clustered RCT is developed around data from previous clustered trials assessing game skill (e.g., support, decision-making and defence) and fundamental movement skill (e.g., throw, kick, under-arm throw and catch) development [23, 24]. The lowest effects in these trails were observed for the fundamental movement skill of catch (effect $d = 0.4$), and the game skill of decision making ($d = 0.4$).

Without accounting for clustering among schools (schools being alike and reducing the power of data), approximately 156 students are required to detect an effect of d = 0.4 at 80% power with alpha 0.05. To adjust for clustering, the following correction factor is applied (1+ (m− 1) x ICC) [25], where m = students per school and ICC = the intra-class correlation coefficient (between school variance / between school variance + within school variance). Assumptions are based on clustering at the school level (one class recruited per school, with ~25 students per class), and a conservative ICC of 0.20 based on data from a school based intervention [22], resulting in a correction factor of 5.8. The resulting student sample is ~904 students at 36 schools (18 intervention, 18 control).

Setting and participants

To align with the typical motor development phase associated with CMS, recruitment of participants will be targeted at children 8–12 years, spread across 4 school years/grades. As this age range straddles two levels of schooling (primary/elementary, secondary/middle) 6 schools from each level at each site will be invited to the trial. Existing university networks will be used to recruit schools and if more than the required number of schools replies positively, schools will be stratified into age groupings prior to random selection (coin toss) of inclusion into the study. Recruitment will result in 3 sites each containing 12 schools (6 experiment, 6 control) evenly distributed across primary/elementary, secondary/middle schools yielding approximately 300 students at each site and a total of ~904 students for the trial. Representatives of each school will be invited to an information event to support their decision-making in becoming involved.

Consent and permissions

Prior to the intervention, parents/carers of the participating students and the Headteacher/Principal of the school, acting as a gatekeeper, will be provided with study information and provide written informed consent. The students will also be provided with an information sheet and assent form, specifically written to respond to their reading age. If the Headteacher agrees to students at their school participating in the intervention, they will need to sign and return the gatekeeper consent form to the research team and distribute information packs to prospective participating students and their parents/carers. If parents/carers permit their child to participate in the intervention they will need to complete, sign, and return the informed consent forms to school teachers along with assent forms from the children. All completed forms will then be collected by the research team from teachers.

Randomization

Following baseline assessments, schools at each site will be ranked by their local socio-economic identifier within schooling level strata (primary/elementary, secondary/middle schools). An individual not involved in the research project will then randomize schools within ranked pairs to either the MOGBA intervention or a wait list control group using a random number generator.

Intervention

MOGBA is presented as a series of 14 resource cards with the front of the card illustrating the game as well as sections describing 'what you need' (equipment), 'set up like this', 'keep it safe' and 'change the game'. On the reverse of the card, there is an assessment framework that illustrates the movement being assessed and provides criteria for the practitioner to use to score the child's performance using two constructs. The assessment has two focal aspects most closely related to the movement being assessed (i.e. two aspecsts from: head, arms, legs or body) with associated criteria, alongside which the numerical values are placed following a child's assessment. Given the complex nature of the assessment and projected groups size (20–25 children), a subset of children will be selected for assessment during each activity.

The resource also contains an 'introductory section' that explains the nature of children's movement development and the purpose of MOGBA, as well as a 'change the challenge' section. 'Change the challenge' provides guidance for practitioners on how to differentiate the activity to meet the diverse needs of children in relation to Goodway, Osmun & Gallahue's [1] notion of Space, Effort and Relationships.

Facilitators recruited by each site will deliver the 14 MOGBA activities in schools over a 9-week period, following two three-hour facilitator development workshops. Facilitators are affiliated to the University and have considerable experience in Physical Education and/or coaching settings. At each site, all 14 MOGBA activities, ranging in categories (Stability (S), Object Control (OC) and Locomotion (L)) and complexity of movement (across three phases) will be delivered, with two sessions each week lasting between 45–60 minutes per session. One MOGBA activity, as presented on the front of the activity resource card and use of the associated assessment protocol will last the duration of the session. An extra four sessions have been accounted for within the intervention period to cater for typical disruptions to timetables. Fig 1 provides an example of the 'Play it' side of a MOGBA activity resource card.

On the reverse of the card, there is an assessment rubric based on various movement frameworks [1, 26], as demonstrated in Fig 2 below. This assessment rubric relies on developmental perspectives of movement competence by establishing the observed child's performance in relation to the movement task, using assessment criteria. Three phases of movement

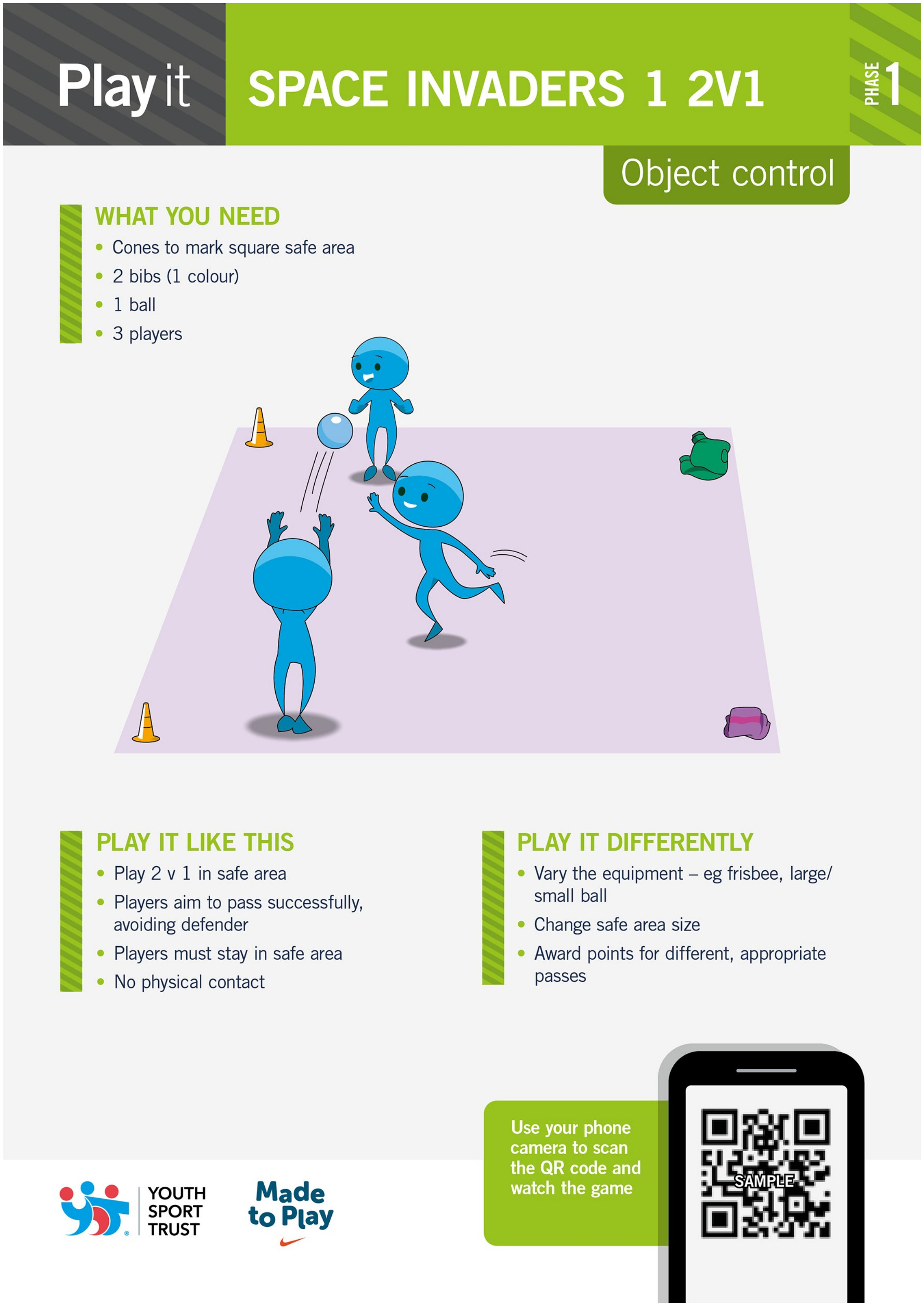

Fig 1. Example of a MOGBA activity (Space Invaders 4, Phase 3, Object Control) front of card 'Play it'.

https://doi.org/10.1371/journal.pone.0253747.g001

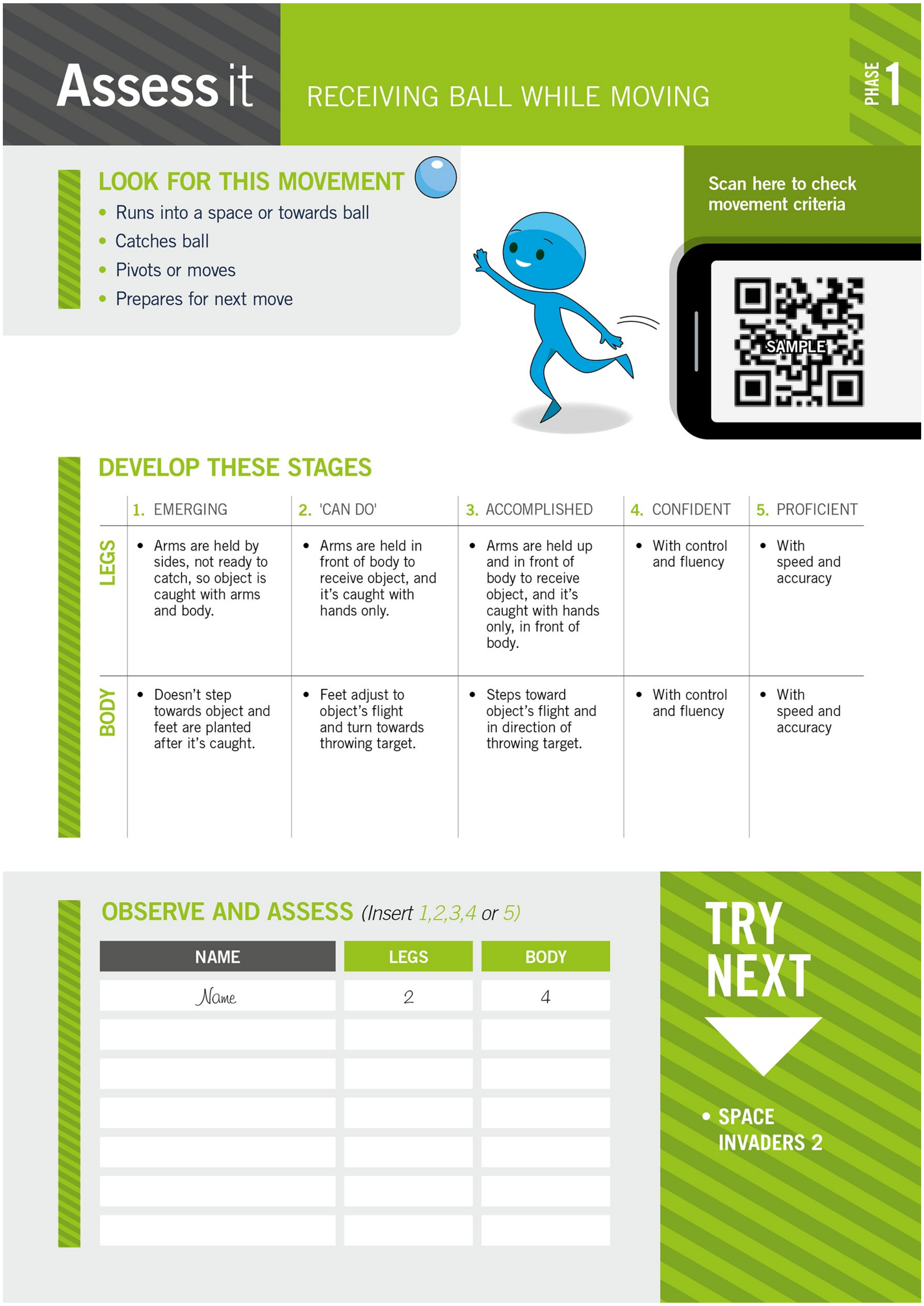

Fig 2. Example of a MOGBA activity (Space Invaders 4, Phase 3, Object Control) reverse of card 'Assess it'.

https://doi.org/10.1371/journal.pone.0253747.g002

competence have been criterion referenced ((1) emerging, (2) can do, (3) accomplished), in a similar way to other movement assessment protocols that have been well received in feasibility trials within similar settings to the intended use of MOGBA [27]. To increase the sensitivity of assessment and negate a potential ceiling effect in scoring the movement task, a further two components of movement competence have been identified for assessment; namely, (1) confidence and (2) proficient. These assessment components have been introduced to encapsulate the broader contextual demands of movement competence and recognize the child's ability to perform the movement tasks with greater degrees of freedom [28, 29].

The MOGBA activities are arranged by Phase (1, 2 and 3) and category of movement (Stability (S), Object control (OC) and Locomotion (L)): Phase 1: 'Space Invaders 1 (2v1)' (OC), 'T-Time' (S), 'Square Ball' (OC) and 'Raid' (L). Phase 2: 'Space Invaders 2 (3v2) (OC), 'Cornerball' (OC), 'End Zone 1' (S), Kabbadi (S), 'Gateways' (L) and 'Potted Skills Circuit' (L). Phase 3: 'Space Invaders 3 (3v4)' (OC), 'Mat Rounders' (OC), 'End zone 2' (S) and 'Tag' (L).

MOGBA activities are designed as innovative, dynamic and fun activities that are non-sport specific and presented as three distinct phases. Phase 1 activities involve simple movement tasks, often performed at individual level and without the complexity associated with the utilization of perceptual-cognitive skills such as anticipation and decision making [30] needed in partner, small group work or team games. This phase is seen as an opportunity for children to use previously acquired FMS in more complex situations, but in a way that supports the transition of a child's movement development from FMS to CMS in a progressive and staged way.

Phase 2 activities build upon the re-introduction of FMS in more complex activities experienced in Phase 1 to further explore the child's ability to refine, adapt, combine and apply FMS in more pressurized environments. There is a continuous theme of space exploration throughout the phases, within the 'Space Invaders' activities that allows children to experience the significance of space in game play in a range of increasingly complex situations.

Phase 3 activities are situated as close to traditional forms of game play as possible, without the constraints of rules and codes associated with a specific game. In phase 3, children are asked to apply learning from Phase 1 and 2 to respond to the demands of the activity and use assessment feedback to improve their performance of movement tasks within the games. This progressive nature of activity development is appropriate given observations of players coupling their actions in both space and time to information unfolding from key environmental and task constraints during performance [31].

Whilst most of the sections within the 'play it' resources are self-explanatory, the 'Change the game' section challenges the practitioner to modify the game to challenge children in different ways. This is based on theories of differentiation that suggests modifying core aspects of activity design will enable children to engage in differing ways that better meet their needs [32].

To further differentiate opportunities for participating children, a 'Change the challenge' section provided separately offers guidance for practitioners on how to differentiate the activity to meet the diverse needs of children in relation to Goodway et al. [1] notion of Space, Effort and Relationships (SER). This section was originally contained within the core activity cards, alongside the illustration of the activity and text supporting the establishment and playing of the game. Findings from the original feasibility trial [33] suggested the amount of detail on the card was overwhelming for some facilitators and feedback indicated that the 'change the challenge' section was not being used in the initial stages of delivery. Consequently, the authors decided to remove this aspect from the main activity card and present this information as a separate section within the resource as something that would be used following the initial establishment of the primary function of the game and assessment protocols. During the intervention, the facilitator modifies the activity using one or more of the SER principles to

challenge the child in different ways within the general construct of the game. This allows for a movement focus to be maintained throughout the development of the game, whilst challenging children at different developmental levels to respond to the variation on the task created by SER.

Quick Response (QR) codes are used to support the ability of the facilitator to acquire the relevant information needed to establish the activity and assessment focus through viewing a video of (a) the activity being played (on the front of the card) and (b) the movement task being assessed (on the back of the card). This level of visual support has been recognised as an effective mode of information dissemination in previous developments of similar resources [34].

The integration of teaching and assessment guidance in a single resource is seldom seen in children's physical education, physical activity or sport contexts. We have designed the resource in this way to encourage facilitators to view teaching, learning and assessment as interdependent aspects of a positive learning environment [35, 36]. Through the provision of activities with an associated assessment protocol it is also hypothesized that facilitators will use assessment data to inform subsequent curriculum design and tailor the choice of activities according to the developmental status of children within the group.

Illustrations used within the resource were designed with the following principles in mind: (i) characters to be gender and ethnicity neutral, (ii) text to be limited to minimal instructions, (iii) characters to be sufficiently human-like to identify changes in head, arms, body and legs to support facilitator recognition of movement changes (iv) use of colour and font-coding to identify which phase of development and movement category was being developed (v) navigation around the information on the card to be easily defined.

Coach workshops

Two, three hour, workshops involving a mixture of theory and practical content, designed by the Principle Investigator and lead investigator from each of the sites (site lead) will be delivered by each site lead, in- country, to four facilitators. The format of the coach learning session will be: (i) the nature of children's movement development, (ii) rationale, structure and purpose of MOGBA, (iii) intervention programme of activities, (iv) changing the challenge, (v) differentiating opportunities (vi) assessing and recording movement competence and (vii) using assessment to guide teaching and improve movement competence.

Fidelity of training

The fidelity of training of the coach delivering MOGBA will be measured through the coach's adherence to a number of key determinants of successful MOGBA delivery, as agreed by the advisory panel. These determinants are listed below in Table 1, which is an adaption of a validated delivery protocol for physical education lessons [37].

Fidelity of intervention

Each site lead will act as a mentor to the facilitators throughout the program, offering feedback that will be framed by the fidelity observational measure used in the training workshops (Table 1). This measure will also be used as a self-reporting mechanism for coaches to reflect upon their delivery after every session and will be used to form dialogue between the mentor and coach within the mentoring process. Mentoring ensures the coaches involved understand the format and purpose of the designed content, and for coaches to observe, undertake, problem solve and trouble shoot more effective practice with an academic partner, in the authentic context of their own coaching sessions [38–40].

Table 1. Fidelity of MOGBA coach training checklist.

Core Principles	OMG a TEST	Core Practices
**Clear game and Skill Introduction & Demonstration**	**O**rganise a group<br>**M**ove students into the GO position for the activity<br>**G**ive a demonstration and a few rules simultaneously (Get it Moving!)	Coach creates the game in an appropriate space, following equipment and health and safety guidelines<br>Begin lessons with a clear statement of the lesson goals (SOL)<br>Reviews prior skills and knowledge (movement focus from previous phases) before beginning instruction<br>Provides direct and clear description of 'How to Play'<br>Checks for understanding
**Targeted Elicitation**	**T**ry the game- resist over instruction, increase activity exposure<br>**E**valuate the game and student performance<br>Uses '**S**TOP' to interject for development of activity or movement quality<br>**T**ransitions students effectively between activities	Children perform the target SOL movements<br>Coach checks for accurate performance and provides feedback<br>Coach uses 'change the game' to ensure optimum engagement
**Repeated Guided Practice**		Coach provides repeated learning and practice opportunities<br>Coach uses SER to differentiate activity<br>Guides with cues (verbal, visual, physical)<br>Provides individualised guided and varied practice opportunities<br>Breaks down complex skills into smaller instructional units, where necessary
**Coach Responsiveness**		Provides individualized support and feedback<br>Shows enthusiasm and is actively engaged with students<br>Maintains class control<br>Promotes high levels of reaching intended outcomes
**Movement assessment**		Coach identifies which children are to be assessed<br>Accurately uses assessment criteria<br>Affords repeated opportunities for assessment
**Child Engagement**		All children are actively participating, little wandering or off task behaviours<br>Children are watching and listening when Coach is instructing<br>Children are focusing their attention on the task and attempting the task as described by the teacher<br>Children show enthusiasm for the activity tasks

https://doi.org/10.1371/journal.pone.0253747.t001

Mentors will provide phone based feedback for the first 2 weeks of the program. All program facilitators will then be observed by their mentor at Weeks 3 and 7 of the 9-week intervention, with sessions referenced against content indicators, as established in Table 1, above. Sessions are also judged by adherence to (1) set-up criteria (2) play the game (3) change the challenge, and (4) assessment of movement, to obtain the percentage of agreement for each of these sets of statements (E.g. lesson agreement with one of four activity based statements = 25% activity agreement). These agreement values are used to indicate: i) if activity delivery at each time point was in line with a movement-based approach, and ii) if the fidelity of the instruction undertaken by the program facilitators was in line with the intended nature of the intervention. A register of attendance at learning sessions for facilitators and at activity sessions for children is maintained to monitor adherence at sessions across the intervention period.

The processes and procedures identified above represent the evaluation of the three big constructs of fidelity [41]: 1) *usability* (the extent to which the user can be trained to deliver MOGBA and is able to implement it), 2) *feasibility* (the extent to which MOGBA can be delivered in authentic settings of schools), and 3) *fidelity of implementation* (the extent to which MOGBA is delivered by end users in authentic contexts of school). In conducting these procedures we get at: 1) dose, 2) adherence, 3) exposure, 4) quality of delivery and 5) participant responsiveness, as well as addressing fidelity for the training of facilitators and the delivery of the intervention.

Outcomes

Movement competence. The primary outcome of the RCT is to improve children's CMS as a result of exposure to a MOGBA program, specifically created for this study. The Dragon Challenge [17] movement assessment protocol will be used to observe and assess the movement competence of children.

Physical fitness. A composite secondary outcome of children's physical fitness will be measured using a standing long jump, plank and height. Standing long jump will be assessed by following the guidance provided in the Assessment of Physical Activity [42] manual. Standing long jump will be scaled by height resulting in a percent of standing height jumped. The plank protocol requires participants to maintain a static prone position with only forearms and toes touching the ground [43]. Participants will be asked to keep their feet together with toes curled under the feet, elbows forearm distance apart, and hands clasped together against the floor mat. Participants will be asked to maintain eye contact with their hands, a neutral spine, and a straight line from head to ankles. The child will be given one 5-s practice trial, during which the examiner will instruct the child to adopt the proper position, followed by a 25-s period of rest. The test will begin when the participant demonstrates the correct position; a stopwatch will be started. Participants will be allowed to deviate from the correct position once and can continue the test if they immediately resume the correct starting position within 3 seconds. The test will be terminated on the second deviation from the correct position or if the participant did not return to the correct position after the first warning; the stopwatch will be stopped and the time taken on the test will be recorded as the test score for the plank. Only one trial of the plank will be allowed.

Height will be measured with participants barefoot and in physical education clothes. Height will be assessed using a Leicester Height Measure (Type Seca; Birmingham, UK; range 14 to 207 cm). Instruments will be calibrated to ensure accurate measures. Additionally, sitting height will be assessed with the participant sitting on the box with his or her back and buttocks to the backboard of the stadiometer, and with his or her head in the Franckfort horizontal plane [44].

MVPA and sedentariness. To examine if MOGBA has an in-program effect on the amount of MVPA students are engaged in at school, in-school MVPA will be measured using ActiGraph GT3X+, wGT3X-BT or Axivity AX3 accelerometers worn on the wrist as part of a 5-day in-school protocol at baseline and between weeks 7–9 of the intervention period. Accelerometers will be fitted by the classroom teacher at the beginning of each school day (~9am), with the devices removed at the end of the school day (~3pm). Additionally, to investigate if MOGBA has an effect on MVPA during PE lessons, analysis will be performed for the specific session times (2 x 45–60 minutes) in which physical education (control) or MOGBA (intervention) is undertaken.

Accelerometer data are downloaded in raw format using Actilife Software (version 6.13.3) and processed in R (http://cran.r-project.org/) using the software package GGIR [45]. Data extracted between the first midnight and the last midnight are retained for the analysis. Non-wear time is classified within a 60 min time window if for at least two out of the three axes, the standard deviation was less than 13 mg and the value range is less than 50 mg [46]. Data are reduced by calculating the average gravity-based acceleration units (g) per 1-s epoch, with daily time spent in moderate-to-vigorous physical activity (MVPA) determined using the sum of epochs averaging above 201 mg [47]. The average minutes spent in MVPA per day and average daily wear time is computed using data from each participant's valid days. A valid day is defined as $\geq$5h on [48], with participants included in the analysis if they have data for at least 4 valid days [49].

Game and physical self-perceptions. An individual's levels of perceived and actual physical competence have differing effects on their levels of motivation in a physical domain.

Among children, perceived competence appears to affect motivation towards PA more than actual competence [50], and perceived competence appears to mediate the relationship between actual motor skill competence and PA levels [51, 52].

Children in most countries are exposed to increasing levels of game play and team sports as part of the school curriculum from ages 9–12 years. Given the positive relationship between motor-skill competence [7, 46, 53] and game play competence [7], assessment of changes in perceived physical competence and specific game-play competence appear relevant within an intervention designed to improve both.

The Game play perception profile (GPPP) is used to assess self-perception of perceptual-cognitive related game play among upper elementary school children. The GPPP measures two distinct factors using a four-point scale (Strongly Disagree—Strongly Agree): i) game-play perception–five items reflecting ability across game themes, and ii) game self-perception–four items reflecting abilities in relation to others. The instrument displays acceptable item distribution, item total correlations (>.30) and internal reliability ($\alpha = .84$ & .85). Change in mean (< 5%) and ICC (.81 & .81) indicate the sub-scales display high levels of test-retest reliability, with face validity (proportion of correctly identified question themes) above 80% for all items and sub-scales. The validity of this measure will be further tested in the recruited cohort using confirmatory factor analysis of the underlying factor structure. Children's physical self-perception will also be measured using an adaptation of Harter's physical self-perception scale [54]. Six items are framed as either/or statements using a two-point scale on either side of the statement (Really true for me, sort of true for me).

Statistical analyses

This trial aims to examine whether MOGBA (a) improves children's movement competence (Dragon Challenge); (b) increases MVPA during PE lessons (acceleromters), (c) reduces sedentary time both in school and outside of school (accelerometers) (d), improves physical fitness (plank and standing long jump), (e) improves self-perceptions of game and physical competence (Game Play Perception Profile). We hypothesize that children in the MOGBA intervention, compared to those in the control group, will display more favorable changes in these variables identified above.

Statistical analyses of the primary and secondary outcomes will be conducted with linear mixed models using IBM PASW Statistics 25 (SPSS Inc. Chicago, IL) software. Impacts are estimated using an intention-to-treat (ITT) approach, with alpha levels set at $p < 0.05$. Linear mixed models will be fitted to compare continuous outcomes. Group, time, and group-by-time interaction will be assessed as fixed effects within the model, with covariates of gender and year level also included as fixed effects. The school a student belongs to and the research site will be included as a random intercepts within the model to account for the multi-level nature of the data, and subject (student ID) will be included as a random intercept to model repeated measures at the individual level. Differences of means and 95% confidence intervals (CIs) will be determined using the linear mixed models. If the variables used for secondary outcomes (questionnaires) fail tests of multivariate normality, non-parametric methods will be utilised, or the variables for use in the linear models will be an individual's factor score from a confirmatory factor analysis of the scales with the appropriate estimator chosen given the non/normal nature of the data.

Discussion

In this paper, we presented the rationale and study protocol for a cluster RCT of the MOGBA intervention. To the authors' knowledge, MOGBA is the first CMS intervention for children

aged 8–12 years in multiple sport settings. MOGBA is also the first intervention that combines delivery and movement competence assessment protocols as well as conducting the assessment within a game situation. Through the establishment of a global RCT, we can begin to assess the effectiveness of MOGBA in affecting children's movement competence, PA and self-perceptions across countries.

Evidence suggests that children are not achieving average levels of movement competence, globally [55] and within the targeted countries within this intervention (England: [15]; Ireland: [56]; Australia: [57]). Evidence also suggests that children are dropping out of organized sport at an unprecedented rate [58, 59]. The reasons for this dropout are varied and complex and include early specialization, in which a child pursues one sport and/or quits other sports to pursue one sport, which favors the development of technical skills in one sport [60]. Other factors involve a loss of focus on fun and an overemphasis on technical and tactical aspects of the game [61]. Our ability to provide positive and supportive bridging experiences for children to continue to develop their movement competence from Fundamental to Complex movement stages seems critical in providing the platform necessary for lifelong participation in sport and physical activity.

Author Contributions

Conceptualization: David Morley, James Rudd, Johann Issartel, Jackie Goodway, Donna O'Connor, Jonathon Foulkes, Mark Babic, Jennifer Kavanagh, Andrew Miller.

Funding acquisition: David Morley.

Methodology: James Rudd, Johann Issartel, Jackie Goodway, Donna O'Connor, Jonathon Foulkes, Mark Babic, Jennifer Kavanagh, Andrew Miller.

Project administration: David Morley, James Rudd, Johann Issartel, Jackie Goodway, Jonathon Foulkes, Jennifer Kavanagh, Andrew Miller.

Supervision: David Morley, James Rudd, Johann Issartel.

Writing – original draft: David Morley, James Rudd, Johann Issartel, Jackie Goodway, Donna O'Connor, Jonathon Foulkes, Mark Babic, Jennifer Kavanagh, Andrew Miller.

References

1. Goodway JD, Ozmun JC, Gallahue DL. *Understanding motor development*: *Infants*, *children*, *adolescents*, *adults*. Jones & Bartlett Publishers; 2019 Oct 15.
2. Haga M, Pedersen AV, Sigmundsson H. Interrelationship among selected measures of motor skills. Child care health dev. 2008 Mar; 34(2):245–8. https://doi.org/10.1111/j.1365-2214.2007.00793.x PMID: 18028473
3. Seefeldt V, Nadeau C, Newell WH, Roberts G. Psychology of motor behavior and sport. Developmental motor patterns: Implications for elementary school physical education. Champaign, IL. Human Kinetics. 1980.
4. Holfelder B, Schott N. Relationship of fundamental movement skills and physical activity in children and adolescents: A systematic review. Psychol Sport Exerc. 2014 Jul 1; 15(4):382–91.
5. Lai SK, Costigan SA, Morgan PJ, Lubans DR, Stodden DF, Salmon J, et al. Do school-based interventions focusing on physical activity, fitness, or fundamental movement skill competency produce a sustained impact in these outcomes in children and adolescents? A systematic review of follow-up studies. Sports Med. 2014 Jan 1; 44(1):67–79. https://doi.org/10.1007/s40279-013-0099-9 PMID: 24122775
6. Bremer E, Cairney J. Fundamental movement skills and health-related outcomes: A narrative review of longitudinal and intervention studies targeting typically developing children. Am J Lifestyle Med. 2018 Mar; 12(2):148–59. https://doi.org/10.1177/1559827616640196 PMID: 30202387
7. Miller A, Eather N, Duncan M, Lubans DR. Associations of object control motor skill proficiency, game play competence, physical activity and cardiorespiratory fitness among primary school children. J

Sports Sci. 2019 Jan 17; 37(2):173–9. https://doi.org/10.1080/02640414.2018.1488384 PMID: 29912626

8. Strong WB, Malina RM, Blimkie CJ, Daniels SR, Dishman RK, Gutin B, et al. Evidence based physical activity for school-age youth. J Pediatr 2005, 146(6):732–737. https://doi.org/10.1016/j.jpeds.2005.01.055 PMID: 15973308
9. van der Fels IM, te Wierike SC, Hartman E, Elferink-Gemser MT, Smith J, Visscher C. The relationship between motor skills and cognitive skills in 4–16 year old typically developing children: A systematic review. J Sci Med Sport. 2015 Nov 1; 18(6):697–703. https://doi.org/10.1016/j.jsams.2014.09.007 PMID: 25311901
10. Burton AW, Miller DE. Movement skill assessment. Human Kinetics; 1998.
11. Robinson LE, Goodway JD. Instructional climates in preschool children who are at-risk. Part I: Object-control skill development. Res Q Exerc Sport. 2009 Sep 1; 80(3):533–42. https://doi.org/10.1080/02701367.2009.10599591 PMID: 19791639
12. Bardid F, Vannozzi G, Logan SW, Hardy LL, Barnett LM. A hitchhiker's guide to assessing young people's motor competence: Deciding what method to use. J Sci Med Sport. 2019 Mar 1; 22(3):311–8. https://doi.org/10.1016/j.jsams.2018.08.007 PMID: 30166086
13. Cools W, De Martelaer K, Samaey C, Andries C. Movement skill assessment of typically developing preschool children: A review of seven movement skill assessment tools. J Sports Sci Med. 2009 Jun; 8 (2):154. PMID: 24149522
14. Giblin S, Collins D, Button C. Physical literacy: importance, assessment and future directions. Sports Med. 2014 Sep 1; 44(9):1177–84. https://doi.org/10.1007/s40279-014-0205-7 PMID: 24898813
15. Morley D, Till K, Ogilvie P, Turner G. Influences of gender and socioeconomic status on the motor proficiency of children in the UK. Hum Mov Sci. 2015 Dec 1; 44:150–6. https://doi.org/10.1016/j.humov.2015.08.022 PMID: 26342797
16. Longmuir PE, Boyer C, Lloyd M, Borghese MM, Knight E, Saunders TJ, et al. Canadian Agility and Movement Skill Assessment (CAMSA): Validity, objectivity, and reliability evidence for children 8–12 years of age. J Sport Health Sci. 2017 Jun 1; 6(2):231–40. https://doi.org/10.1016/j.jshs.2015.11.004 PMID: 30356598
17. Tyler R, Foweather L, Mackintosh KA, Stratton G. A dynamic assessment of children's physical competence: The dragon challenge. Med Sci Sports Exerc. 2018 Dec; 50(12):2474. https://doi.org/10.1249/MSS.0000000000001739 PMID: 30067588
18. Lander N, Morgan PJ, Salmon J, Barnett LM. Teachers' perceptions of a fundamental movement skill (FMS) assessment battery in a school setting. Meas Phys Educ Exerc Sci. 2016 Jan 2; 20(1):50–62.
19. Lander N, Morgan PJ, Salmon J, Logan SW, Barnett LM. The reliability and validity of an authentic motor skill assessment tool for early adolescent girls in an Australian school setting. J Sci Med Sport. 2017 Jun 1; 20(6):590–4. https://doi.org/10.1016/j.jsams.2016.11.007 PMID: 28131506
20. Ng JL, Button C. Reconsidering the fundamental movement skills construct: Implications for assessment. Mov Sport Sci. 2018(4):19–29.
21. Nike Global and Social Community Impact. 2019. Available from: https://communityimpact.nike.com/made-to-play
22. Dobbins M, Husson H, DeCorby K, LaRocca RL. School-based physical activity programs for promoting physical activity and fitness in children and adolescents aged 6 to 18. Cochrane Database of Syst Rev. 2013(2). https://doi.org/10.1002/14651858.CD007651.pub2 PMID: 23450577
23. Miller A, Christensen EM, Eather N, Sproule J, Annis-Brown L, Lubans DR. The PLUNGE randomized controlled trial: Evaluation of a games-based physical activity professional learning program in primary school physical education. Prev Med. 2015 May 1; 74:1–8. https://doi.org/10.1016/j.ypmed.2015.02.002 PMID: 25668220
24. Miller A, Harvey S, Morley D, Nemes R, Janes M, Eather N. Exposing athletes to playing form activity: Outcomes of a randomised control trial among community netball teams using a game-centred approach. J Sport Sci. 2017 Sep 17; 35(18):1846–57. https://doi.org/10.1080/02640414.2016.1240371 PMID: 27719107
25. Donner A, Klar N. Cluster randomization trials. Stat Methods Med Res. 2000 Apr 1; 9(2):79.
26. Graham C, Holt-Hale SA, Parker M. Determining generic levels of skill proficiency. In: Children moving: A reflective approach to teaching physical education, 10th edition. New York: McGraw-Hill; 2020. pp. 58–66.
27. Foweather L, Van Rossum T, Richardson DJ, Hayes S, Morley D. Primary teachers' recommendations for the development of a teacher-oriented movement assessment tool for 4–7 year children. Meas Phys Educ Exerc Sci. 2018 Dec 3; 23(2):124–34.

28. Bartenieff I, Lewis D. Body movement: Coping with the environment. New York: Gorden & Breach; 1980.
29. Langton TW. Applying Laban's movement framework in elementary physical education. J Phys Educ Recreat Dance. 2007 Jan 1; 78(1):17–53.
30. Williams AM, Ford PR. Expertise and expert performance in sport. Int Rev Sport Exerc Psych. 2008 Mar 1; 1(1):4–18.
31. Travassos B, Araújo D, Duarte R, McGarry T. Spatiotemporal coordination behaviors in futsal (indoor football) are guided by informational game constraints. Hum Mov Sci. 2012 Aug 1; 31(4):932–45. https://doi.org/10.1016/j.humov.2011.10.004 PMID: 22672740
32. Chow JY, Davids K, Button C, Renshaw I. Nonlinear pedagogy in skill acquisition: An introduction. Routledge; 2015 Dec 14.
33. Morley D, van Rossum T. Development of a Movement Oriented Games Based Assessment (MOGBA) resource for 8–12 year old children within Nike's 'Made to Play' portfolio. UK and USA testing. 2019. Available from: https://www.researchgate.net/project/Developing-a-Movement-Oriented-Games-Based-Assessment-MOGBA-for-8-12-year-olds-on-programs-within-Nikes-Made-to-Play-initiative
34. Mehendale D, Masurekar R, Nemade S, Shivthare S. To study the use of QR code in the classroom to enhance motivation, communication, collaboration and critical thinking. Int J Innovat R Comp Comm Eng. 2017; 5(4):6987–93.
35. Newton PE. Clarifying the purposes of educational assessment. Assess Educ. 2007 Jul 1; 14(2):149–70.
36. Gardner J, editor. Assessment and learning. Sage; 2012 Mar 5.
37. Miller A, Eather N, Gray S, Sproule J, Williams C, Gore J, et al. Can continuing professional development utilizing a game-centred approach improve the quality of physical education teaching delivered by generalist primary school teachers?. Eur Phys Ed Rev. 2017 May; 23(2):171–95.
38. Brian A, Goodway JD, Logan JA, Sutherland S. SKIPing with teachers: An early years motor skill intervention. Phys Educ Sport Pedagogy. 2017 May 4; 22(3):270–82.
39. Rhodes C, Beneicke S. Professional development support for poorly performing teachers: challenges and opportunities for school managers in addressing teacher learning needs. J In-service Educ. 2003 Mar 1; 29(1):123–40.
40. Kennedy A. Models of continuing professional development: A framework for analysis. J In-service Educ. 2005 Jun 1; 31(2):235–50.
41. Durlak JA, DuPre EP. Implementation matters: A review of research on the influence of implementation on program outcomes and the factors affecting implementation. Am J Community Pschol. 2008 Jun 1; 41(3–4):327. https://doi.org/10.1007/s10464-008-9165-0 PMID: 18322790
42. Ruiz JR, Castro-Piñero J, España-Romero V, Artero EG, Ortega FB, Cuenca MM, et al. Field-based fitness assessment in young people: the ALPHA health-related fitness test battery for children and adolescents. Br J Sports Med. 2011 May 1; 45(6):518–24. https://doi.org/10.1136/bjsm.2010.075341 PMID: 20961915
43. Strand SL, Hjelm J, Shoepe TC, Fajardo MA. Norms for an isometric muscle endurance test. J Jum Kinet. 2014 Mar 1; 40(1):93–102. https://doi.org/10.2478/hukin-2014-0011 PMID: 25031677
44. Fredriks AM, van Buuren S, Van Heel WJ, Dijkman-Neerincx RH, Verloove-Vanhorick SP, Wit JM. Nationwide age references for sitting height, leg length, and sitting height/height ratio, and their diagnostic value for disproportionate growth disorders. Arch Dis Child. 2005 Aug 1; 90(8):807–12. https://doi.org/10.1136/adc.2004.050799 PMID: 15863466
45. van Hees VT, Gorzelniak L, Leon EC, Eder M, Pias M, Taherian S, et al. Separating movement and gravity components in an acceleration signal and implications for the assessment of human daily physical activity. PloS one. 2013 Apr 23; 8(4):e61691. https://doi.org/10.1371/journal.pone.0061691 PMID: 23626718
46. Sabia S, van Hees VT, Shipley MJ, Trenell MI, Hagger-Johnson G, Elbaz A, et al. Association between questionnaire-and accelerometer-assessed physical activity: the role of sociodemographic factors. Am J Epidemiol. 2014 Mar 15; 179(6):781–90. https://doi.org/10.1093/aje/kwt330 PMID: 24500862
47. Hildebrand MV, VAN VH, Hansen BH, Ekelund UL. Age group comparability of raw accelerometer output from wrist-and hip-worn monitors. Med Sci Sports Exerc. 2014 Sep; 46(9):1816–24. https://doi.org/10.1249/MSS.0000000000000289 PMID: 24887173
48. Barnett LM, Ridgers ND, Salmon J. Associations between young children's perceived and actual ball skill competence and physical activity. J Sci Med Sport. 2015 Mar 1; 18(2):167–71. https://doi.org/10.1016/j.jsams.2014.03.001 PMID: 24685052

49. Trost SG, Mciver KL, Pate RR. Conducting accelerometer-based activity assessments in field-based research. Med Sci Sports Exerc. 2005 Nov 1; 37(11):S531–43. https://doi.org/10.1249/01.mss.0000185657.86065.98 PMID: 16294116

50. Harter S. Effectance motivation reconsidered. Toward a developmental model. Hum Dev. 1978; 21 (1):34–64.

51. Barnett LM, Morgan PJ, van Beurden E, Beard JR. Perceived sports competence mediates the relationship between childhood motor skill proficiency and adolescent physical activity and fitness: a longitudinal assessment. Int J Behav Nutr Phys Act. 2008 Dec; 5(1):1–2. https://doi.org/10.1186/1479-5868-5-40 PMID: 18687148

52. Barnett LM, Morgan PJ, Van Beurden E, Ball K, Lubans DR. A reverse pathway? Actual and perceived skill proficiency and physical activity. Med Sci Sports Exerc. 2011 May 1; 43(5):898–904. https://doi.org/10.1249/MSS.0b013e3181fdfadd PMID: 20962694

53. Lubans DR, Morgan PJ, Cliff DP, Barnett LM, Okely AD. Fundamental movement skills in children and adolescents. Sports Med. 2010 Dec 1; 40(12):1019–35. https://doi.org/10.2165/11536850-000000000-00000 PMID: 21058749

54. Harter S. The perceived confidence scale for children. Child dev, 87–97.

55. Stodden DF, Goodway JD, Langendorfer SJ, Roberton MA, Rudisill ME, Garcia C, et al. A developmental perspective on the role of motor skill competence in physical activity: An emergent relationship. Quest. 2008 May 1; 60(2):290–306.

56. Behan S, Belton S, Peers C, O'Connor NE, Issartel J. Moving Well-Being Well: Investigating the maturation of fundamental movement skill proficiency across sex in Irish children aged five to twelve. J Sport Sci. 2019 Nov 17; 37(22):2604–12. https://doi.org/10.1080/02640414.2019.1651144 PMID: 31379260

57. Hardy L, King L, Espinel P, Cosgrove C, Bauman A. NSW schools physical activity and nutrition survey (SPANS) 2010. 2013 May 20.

58. Fraser-Thomas J, Côté J, Deakin J. Understanding dropout and prolonged engagement in adolescent competitive sport. Psychol Sport Exerc. 2008 Sep 1; 9(5):645–62.

59. Eime RM, Harvey JT, Charity MJ. Sport drop-out during adolescence: is it real, or an artefact of sampling behaviour? Int J Sport Policy Politics. 2019 Oct 2; 11(4):715–26.

60. Myer GD, Jayanthi N, Difiori JP, Faigenbaum AD, Kiefer AW, Logerstedt D, et al. Sport specialization, part I: does early sports specialization increase negative outcomes and reduce the opportunity for success in young athletes? Sports Health. 2015 Sep; 7(5):437–42. https://doi.org/10.1177/1941738115598747 PMID: 26502420

61. Visek AJ, Achrati SM, Mannix HM, McDonnell K, Harris BS, DiPietro L. The fun integration theory: toward sustaining children and adolescents sport participation. J Phys Act Health. 2015 Mar 1; 12 (3):424–33. https://doi.org/10.1123/jpah.2013-0180 PMID: 24770788
